# Human-centred design in industry 4.0: case study review and opportunities for future research

**DOI:** 10.1007/s10845-021-01796-x

**Published:** 2021-06-11

**Authors:** Hien Nguyen Ngoc, Ganix Lasa, Ion Iriarte

**Affiliations:** Design Innovation Center (DBZ), Mondragon Unibertsitatea - Faculty of Engineering, Loramendi, 4, 20500 Arrasate-Mondragón, Gipuzkoa Spain

**Keywords:** Human-centred design, Industry 4.0, Case study review, Research opportunities

## Abstract

The transition to industry 4.0 has impacted factories, but it also affects the entire value chain. In this sense, human-centred factors play a core role in transitioning to sustainable manufacturing processes and consumption. The awareness of human roles in Industry 4.0 is increasing, as evidenced by active work in developing methods, exploring influencing factors, and proving the effectiveness of design oriented to humans. However, numerous studies have been brought into existence but then disconnected from other studies. As a consequence, these studies in industry and research alike are not regularly adopted, and the network of studies is seemingly broad and expands without forming a coherent structure. This study is a unique attempt to bridge the gap through the literature characteristics and lessons learnt derived from a collection of case studies regarding human-centred design (HCD) in the context of Industry 4.0. This objective is achieved by a well-rounded systematic literature review whose special unit of analysis is given to the case studies, delivering contributions in three ways: (1) providing an insight into how the literature has evolved through the cross-disciplinary lens; (2) identifying what research themes associated with design methods are emerging in the field; (3) and setting the research agenda in the context of HCD in Industry 4.0, taking into account the lessons learnt, as uncovered by the in-depth review of case studies.

## Introduction

A challenge of manufacturing today is adapting to an increasingly fluctuating environment and diverse changes to meet the demands of the market. Product life cycles are getting shorter while production batch sizes are getting smaller with dynamic product variants associated with increasing complexity, which is challenging the traditional production systems (Benabdellah et al., 2019; Kuhnle et al., 2021; Ma et al., 2017; Prinz et al., 2019; Windt et al., 2008; Zhu et al., 2015). To manage these dynamics, the industrial concept of Industry 4.0 has come about and has been accepted in both research and industry, a trend linked to digitalization and smart systems that could enable factories to achieve higher production variety with reduced downtimes while improving yield, quality, safety, and decreasing cost and energy consumption (García-Magro & Soriano-Pinar, 2019; Järvenpää et al., 2019; Napoleone et al., 2020; Oztemel & Gursev, 2020; Park & Tran, 2014). Although the adoption of Industry 4.0 in manufacturing reveals positive outcomes, the increased complexity as a collateral effect has also brought many challenges (Bednar & Welch, 2020; Cohen et al., 2019; Fernandez-Carames & Fraga-Lamas, 2018; Mourtzis et al., 2018; Wittenberg, 2015). One of the challenges is to put humans properly at the centre of smart manufacturing design (Grandi et al., 2020; Pacaux-Lemoine et al., 2017; Paelke et al., 2015; Peruzzini et al., 2019; Varshney & Alemzadeh, 2017). An approach to address this challenge is known as HCD. According to International Organization for Standardization (2019), HCD is a multidisciplinary approach incorporating human factors and ergonomics knowledge and techniques to make systems usable. However, the design complexity in smart systems can occur in both directions, where in one direction the human must be able to effectively cooperate with other existing physical system components and simultaneously exchange data with system informatics for hybrid decision making (Fernandez-Carames & Fraga-Lamas, 2018; Schulze et al., 2005; Zheng et al., 2018). The reverse direction is that the design of such smart systems must be capable of sensing and responding to the trust levels of humans they interact with in order to result in more productive relationships between the human and other smart components (Chang et al., 2017; Rogers et al., 2019; Seitz et al., 2021; Song et al., 2016; Van Acker et al., 2020).

Numerous contributions have been written on Industry 4.0 areas; however, the majority of them focus on the technical aspects in which human factors are commonly underestimated (Bhamare et al., 2020; Grandi et al., 2020; Pacaux-Lemoine et al., 2017; Peruzzini et al., 2019; Theuer et al., 2013). There is an increasing concern about how human factors are barely considered in design for products and/or services and poorly addressed in manufacturing, causing complex problems with often unknown consequences across different industrial contexts: nuclear accidents (Wu et al., 2016), market failures in new product development (García-Magro & Soriano-Pinar, 2019), robotic-surgery-related adversities (Varshney & Alemzadeh, 2017), technological accidents during machine manipulation (Pacaux-Lemoine et al., 2017), and interaction issues among humans and smart systems (Jung et al., 2017; Rogers et al., 2019; Streitz, 2019).

The phenomenon of Industry 4.0 reflects contemporary design contexts that frequently contain complex interdependencies of human and non-human actors—internet of thing (IoT) devices, digital and physical environments—shaping the framework of human roles and socio-technical systems (Cimini et al., 2020; Coulton & Lindley, 2019; Jwo et al., 2021; Kong et al., 2019; Kymäläinen et al., 2017). However, this does not mean that the existing concepts of design—for example, design for manufacturing and assembly (Favi et al., 2021), or a traditional design process that considers existing solutions to fulfil the needs of the largest group (Lorentzen & Hedvall, 2018)—are redundant. They have evolved and enlarged the scope of design: manufacturability fosters the collaboration of design and manufacturing operations, taking the perspectives of efficiency, effectiveness and economics into account (Chen et al., 1995; Venkatachalam et al., 1993); social sustainability addresses design for quality of human life by considering transdisciplinary relationships with human diversity (Demirel & Duffy, 2013; Martin et al., 2013; Papetti et al., 2020). These new requirements have impacted the factories themselves, but they affect the entire value chain, from the product design and development process through market segmentation to manufacturing and product disposal management (Bauer et al., 2019; Kong et al., 2019; Pereira Pessôa & Jauregui Becker, 2020). In this sense, for transitioning to sustainable manufacturing processes and consumption, human-centred factors play a core role in the achievement of sustainability-oriented operations throughout the supply chain (Bednar & Welch, 2020; Ceccacci et al., 2019; Grandi et al., 2020; Gualtieri et al., 2020; Lin, 2018; Rossi & Di Nicolantonio, 2020).

To address human-related roles in the context of Industry 4.0, there is a constantly growing interest in research and industrial practices where humans are placed at the centre of design across disciplines. This is manifest in the substantial body of literature providing signposts of theoretical frameworks and models, implementation methodologies, and case studies in cross-disciplinary contexts. The scope of the research is extensive: customer-centric business models associated with customer involvement in design (Adrodegari & Saccani, 2020; Grieger & Ludwig, 2019; Saha et al., 2020; Santos et al., 2018); smart design engineering in which the users and emotional interactions are empowered (Benabdellah et al., 2019; Pereira Pessôa & Jauregui Becker, 2020); technology design in which users are centred (Chen & Duh, 2019; Rogers et al., 2019); interaction designs among operators and smart manufacturing components (Klumpp et al., 2019; Rossi & Di Nicolantonio, 2020); human-centred designs for product development (Chen et al., 2016; Wu et al., 2013); data processing by which humans remain the first design consideration of a data-driven approach (Crabtree & Mortier, 2015; Victorelli et al., 2020b); sustainability in social-technical manufacturing contexts, including social robotic interactions with humans (Bednar & Welch, 2020; Leng & Jiang, 2017; Richert et al., 2018; Streitz, 2019).

Even though a wide array of studies has been created and published, these studies have become disconnected from other studies after publication. As a consequence, these studies in industry and research alike are not regularly adopted, while the network of studies is scattered and diffused without forming any comprehensive structure. Although numerous review papers portrayed the key developments regarding HCD over recent years, they focused on the reflection of emerging trends based on bibliometric results, debates, and priorities in their own research scope with their defined disciplines. Recently, Zarte et al. (2020) conducted SLR to structure design principles for HCD while Victorelli et al. (2020a) provided an understanding of human-data integration with bibliometric analysis. Other representative review studies include Benabdellah et al. (2019), Duque et al. (2019), Kadir et al. (2019), Bazzano et al. (2017). However, the current work does not pay attention to publications whose case studies contain a tremendous source of useful information. The results of a case study can have a very high impact on exploring in-depth conceptual testing and refinement associated with lessons learnt (Kadir et al., 2019; Tetnowski, 2015; Williams, 2011; Yin, 2018), something that deserves to be treated as a special unit of analysis in the review process. Moreover, the review papers also pointed out their own methodological limitations, leading to the call for future research priorities in identifying and deepening the research outcomes of HCD through the cross-disciplinary lens.

To take the perspective of HCD under the transition to Industry 4.0 and simultaneously respond to said call, we contribute to the research through a rigorous review of case studies—to capture the lessons learnt—that have been conducted so far in the literature. The objective is to pave the way for the ongoing developments around the concept and also explain its journey in a systematic and well-rounded methodology. To achieve this objective, we review the existing scientific body of knowledge by:

- providing insight into how the literature has evolved through the cross-disciplinary lens
- identifying what research themes associated with design methods are emerging in the field
- setting the research agenda in the context of HCD in Industry 4.0, taking into account the lessons learnt, as uncovered by the in-depth review of case studies

To achieve the above and contribute to the body of knowledge regarding the HCD domain, this article begins with HCD’s fundamental concepts, which indicate for researchers diverse perspectives on HCD across the value chain in the context of Industry 4.0. The next section presents a strict protocol of SLR that ensures a sufficient amount of quality publications for the analysis. "Literature characterization of human-centred design in industry 4.0" section digs into the literature to unfold the characteristics of HCD. Subsequently, the in-depth review expresses important facts of HCD in the context of Industry 4.0: emerging research schemes among concepts of HCD, diverse design methods and lessons learnt. This article concludes with a comparative discussion of the papers and suggests opportunities for further research.

## Human-centred design in industry 4.0

Nowadays, the fourth industrial revolution develops highly connected resources, integrates smart components and enables interoperability in cyber-physical systems (CPSs) in the twenty-first century (Campbell 2021; Cruz Salazar et al., 2019; Derigent et al., 2020; Duque et al., 2019; Pereira Pessôa & Jauregui Becker, 2020). The changes that trigger Industry 4.0 have impacted different domains throughout the value chain. First, an autonomous system—embedding smart components in CPSs equipped with autonomous capability—achieves a specified goal independently without any human intervention (Gamer et al., 2020; Park & Tran, 2014). However, human intelligence and intervention remain a key role because of the safety, security, social aspects and uncertainties posed by such autonomous systems (Fosch-Villaronga et al., 2020; Gil et al., 2019; Nahavandi, 2017; Santoni de Sio & van den Hoven 2018; Weichhart et al., 2019). Along with advanced technologies in such smart systems, the role of humans has changed and shifted from low-level operations—which can be dangerous, dirty, difficult, and dull tasks—to high expertise and safe tasks (Bauer et al., 2019; Campbell 2021; Nahavandi, 2017; Zhang et al., 2017). This phenomenon highlights two different concepts of HCD: *human-in-the-loop* and *human-on-the-loop systems* (HioTL). The human-in-the-loop system is a system in which a machine executes a task for a specific command and then stops for the human order before continuation. On the other hand, the human-on-the-loop system is an autonomous system that executes a task independently and completely, while the role of humans can provide expertise not available to the system and can respond to issues that the system is unaware of (Kong et al., 2019; Nahavandi, 2017; Richter et al., 2018; Streitz, 2019; Vanderhaegen, 2019). An autonomous system should not imply the exclusion of the human, but it should allow for a seamless integration of humans in both operational levels of the process monitoring and strategic levels of orchestration in the aggregate plan. This approach enables high levels of human collaboration to achieve the common key performance indicators of manufacturing while meeting internal constraints (Gervasi et al., 2020; Pacaux-Lemoine et al., 2017).

In addition, the smart robots work safely with humans in collaborative production systems to autonomously and seamlessly perform collaborative tasks working towards common goals (Boschetti et al., 2021; Cohen et al., 2019; Gervasi et al., 2020; Wojtynek et al., 2019). These collaborative robots, often called *cobots*, relieve the factory workers from the low-level tasks to work side-by-side with humans in order to increase the workstation performance: production pace, efficiency, and higher throughput. In this context, design for the collaboration is well known as *human–robot collaboration* (HRC), which is also interchangeably called *human–robot interaction* (Cohen et al., 2019; Gervasi et al., 2020). Beyond the physical interactions, the collaboration design also enables the robots and humans to share knowledge and learn from others, and so work towards social sustainability, i.e., discussions and accommodation with others’ perspectives (Fosch-Villaronga et al., 2020; Gualtieri et al., 2020; Richert et al., 2018; Weichhart et al., 2019).

In addition to smart systems and cobots, the industry and research alike pose new requirements and means of interactive interfaces among human and non-human actors (e.g., machines, smart devices) to deal with the new challenges: interdependent interactions with complex information, and natural and intuitive communication (Diegel et al., 2004; Haslgrubler et al., 2018; Ong et al., 2020; Weichhart et al., 2019). In the earlier development, the information systems interfaces are usually designed by the technology-oriented approach that adapts humans to the equipment. This lack of consideration of the human results in lower-than-expected manufacturing system performance and an increasing possibility of error rates (Chen & Duh, 2019; Oborski, 2004; Wu et al., 2016). Therefore, putting humans at the centre of interface design is the concept of the *human–machine interface* (HMI), which allows humans to understand and operate a machine in a digital manufacturing context. Design for HMI requires a transdisciplinary approach that takes various disciplines into account: cognitive psychology, industrial design, information processing graphics, human factors, and ergonomics (Oborski, 2004; Ong et al., 2020; Wu et al., 2016).

Beyond industrial applications, the user-friendly design of HMI is important in various domains—desktop, web engineering, and services—with which its application boundary is very blurred (Chang & Lee, 2013; Chang et al., 2017; Hoffmann et al., 2019). Basically, one of the key measurements to understand the degree to which the design of HMI meets usage requirements is its usability, which focuses on functional indicators: usefulness, efficiency, effectiveness, and the learning curve of the user interface. The deeper concept of user multidimensional experience—which considers users’ emotional and psychological responses—is getting increasing attention and is also known as the core concept of *user-centred design* (UCD) (Chen, 2016; Kymäläinen et al., 2017; Lin, 2018; Paelke et al., 2015; Zheng et al., 2018). UCD, also interchangeably called *user-centrality*, embraces the user’s needs and involvement as the centre of the co-designing development process (Mazali, 2018; Wu et al., 2016) in order to enhance user acceptability and acceptance. While the former is a prior mental representation that users have before interacting with a product and/or service, the latter is an evaluation after a real interaction with the design has taken place (Van Acker et al., 2020).

From the perspective of life-cycle design, the increasing variability of products and varying expectations of customers have impacted development and manufacturing at different stages, requiring new solutions that enhance the value of the customer’s interaction with the product along its life cycle (Benabdellah et al., 2019; Chaudhuri et al., 2019; Fernandez-Carames & Fraga-Lamas, 2018; Pezzotta et al., 2018; Zhu et al., 2015). In this evolving scenario, manufacturers navigate from product-oriented development to the servitization phenomenon in which the concept of *product-service systems* (PSS) is a result of product and service integration. PSS is capable of fulfilling the customer’s present requirements while being adaptable to future needs and necessities through all their life-cycle stages (Cheah et al., 2019; Haber & Fargnoli, 2019; Leoni, 2019; Mourtzis et al., 2018; Pezzotta et al., 2018; Zhu et al., 2015). PSS requires a human-centred design thinking process that not only generates the value-in-use to the customer through the identification of the latent requirements, but also manages the stakeholders and the technical feasibility (Cheah et al., 2019; Santos et al., 2018). The approach of HCD, such as service design, plays an important role in the design of service-oriented value propositions by providing a set of methods to improve customer experience and understand emerging social trends (Iriarte et al., 2018).

The value chain itself is being reconfigured because the type of value exchange is shifted from selling products to providing services in order to optimize competitiveness through market segmentation strategies towards customer personalization. Smart PSS allows for a completely new relationship between manufacturers and customers and thus enables new business models towards *customer-centricity* that facilitate customer-focused and co-creation relationships towards sustainability for business, customers, and stakeholders (Anke, 2019; Bednar & Welch, 2020; Benabdellah et al., 2019; Grieger & Ludwig, 2019; Ma et al., 2017; Saha et al., 2020). This phenomenon is enabled by the ubiquity of digital technologies that allows for a fundamental shift in the business landscape in which the individual customer is at the centre of design activities, at the point of origin, and an active participant across different business processes: innovation, development, management, and production to deliver “smartness” values (Brenner et al., 2014; Mazali, 2018; Zheng et al., 2018).

*Smartness* is a socio-technical phenomenon—in which the production processes and the products themselves are technical aspects—that impacts society’s awareness of sustainability in terms of the environmental, social, and economic aspects (Bednar & Welch, 2020; Fu et al., 2019; Gualtieri et al., 2020; Pereira Pessôa & Jauregui Becker, 2020). There will be a need for a strategic balance between shorter- and longer-term desires, values, and policies, and the interests of different groups of stakeholders. Technology alone cannot give an organization a competitive edge or provide an industry step change, but an organization must be sustainable and have an architecture based on financial, ecological, and socio-technical systems. This context reconfigures the interrelationship among human and non-human actors: people and organizations, technologies and manufacturing systems, and production and consumption. Smartness expresses a new relationship between society and technology in the name of Industry 4.0 (Bauer et al., 2019; Bednar & Welch, 2020; Mazali, 2018; Rogers et al., 2019; Rossi & Di Nicolantonio, 2020; Yao et al., 2019).

The advent of Industry 4.0 has made many changes, and the concepts of design oriented to humans are not exceptional. Some concepts are defined in different contexts, and the boundaries of their application overlap and are often used interchangeably. The similarity among these concepts is a multi-objective approach that aims at designing products and/or services towards human well-being while ensuring sustainable development. In a broader sense, this multi-objective approach addresses not only human factors and ergonomics towards human diversity, but also design for manufacturability: the design process must be efficient; the manufacturing processes must be capable, proactive, and economic (Anderson, 2014; Favi et al., 2021; Sinclair, 1992). This perspective must also take the approach of life-cycle management that aims at managing the activities of products and/or services across the life cycle towards sustainability, such as life-cycle cost analysis for economics (Aurich et al., 2007; Jasiulewicz-Kaczmarek et al., 2021; Kambanou, 2020). This multi-objective approach in HCD is not only consistent with the definition of HCD reported by International Organization for Standardization (2019) (Fernandez-Carames & Fraga-Lamas, 2018; Rossi & Di Nicolantonio, 2020) but also provides a broader perspective throughout the value chain in the context of Industry 4.0.

Due to the broader perspective and diverse contexts in which the concepts regarding HCD have emerged and spread across disciplines, it would be difficult for scholars to set a proper research direction. This difficulty motivates us to review and structure lessons learnt in literature via the cross-disciplinary lens to identify coherent research directions for subsequent researchers and industrial practitioners alike. To realize our objective, the following section presents the protocol of SLR that allows the body of knowledge to be gathered in a systematic but objective way.

## Research methodology

**Fig. 1:**
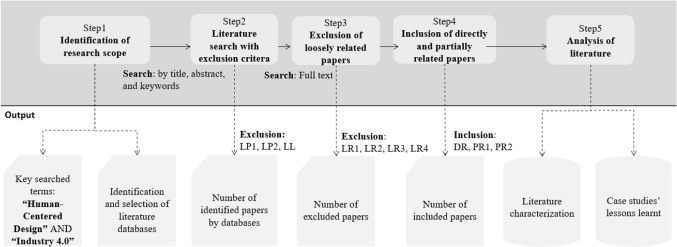
A process flow of systematic literature review

Figure 1 shows a process flow of SLR whose objective is to sufficiently cover the research topic and provide evidence with minimization of subjectivity and bias (Boell & Cecez-Kecmanovic, 2015; Tranfield et al., 2003).

First, there are two fundamental keywords, including “human-centered design” and “industry 4.0”. However, scholars use disparate terms to describe the concepts, and the boundaries of these concepts remain blurred, as analysed in "Human-centred design in industry 4.0" section. Therefore, a wide range of keywords were identified and combined to discover comprehensively and objectively across a broad range of well-known databases whose description is provided by “Appendix” (Table 9): Web of Science, Scopus, Science Direct, Emerald, SpringerLink, Engineering Village, SEGA Journals, and EBSCO. Covering a wide range of substantial databases is one of the decisive efforts for overcoming the limitations of a single database, as reported by Saha et al. (2020). One problem with this breadth of databases is the noticeable difference among their search functionality that requires adjustment according to each database, as detailed by “Appendix” (Table 10).

As a result, there are 265 identified papers, and nearly 162 of them are found by the database of SpringerLink and Emerald, whose disciplines focus on varying fields—science, technology, engineering, and management—that show the transdisciplinary applications of HCD. Table 1 also shows that the number of papers found across databases decreases while that of duplicate papers among them increases proportionally, which shows that papers relevant to this research have been sufficiently covered and reached a state of saturation.

**Table 1:** Identified papers by database

Searching database	Identified papers	Duplicate papers	Non-duplicate papers
SpringerLink	106	1	105
Emerald	56	1	55
Web of Science	14	0	14
Scopus	25	11	14
SAGE Journals	11	1	10
ScienceDirect	17	8	9
EBSCO	25	18	7
Engineering Village	11	10	1
Total	265	50	215

The next step continues with the review protocol to distinguish two groups of inclusion and three groups of exclusion criteria associated with their corresponding description, described in Table 2. In addition to the exclusion of duplicate papers (LP2), we also ensure the credibility of published papers by excluding papers that have not undergone a review process and have been published in journals (LP1).

**Table 2:** Inclusion and exclusion criteria

I/E	Criteria	Coded	Description	Identified papers
			Total identified papers	265
Inclusion			Total included papers	77
	Directly related	DR	An abstract indicates that the full text of the article is *directly* dedicated to HCD *and* Industry 4.0 *in* the context of manufacturing	24
	Partially related	PR1	An abstract indicates that the full text of the article is *directly* dedicated to HCD *and* Industry 4.0 *beyond* the context of manufacturing	6
		PR2	An abstract indicates HCD *and* Industry 4.0, but the full text only provides discussions on one or some aspects of HCD	47
Exclusion			Total excluded papers	188
	Loosely related	LR1	HCD *and* Industry 4.0 are only mentioned as an example	3
		LR2	HCD *and* Industry 4.0 are only mentioned as a part of its future research direction, future perspective or future requirement	5
		LR3	HCD *and* Industry 4.0 are only mentioned as a cited expression	2
		LR4	HCD *and* Industry 4.0 are only mentioned in keywords and/or references	103
	Limited publication	LP1	A paper is not published as a journal article in the studied databases	24
		LP2	A paper is duplicated on the different studied databases	50
	Limited language	LL	A full-text paper is not mainly written in English	1

Given our competence in the language, the papers written in non-English language (LL) are not considered for this study. To keep our research focus, we also excluded all irrelevant papers that mention HCD and Industry 4.0 as examples (LR1) instead of their main research subject; mention the research agenda (LR2) instead of research focus; or cite expressions (LR3), keywords and/or references (LR4). For instance, we found the paper published by Ribeiro and Bjorkman (2018), “Transitioning From Standard Automation Solutions to Cyber-Physical Production Systems: An Assessment of Critical Conceptual and Technical Challenges”, as the search result on the database of Web of Science. However, the paper focuses on the aspects of CPSs instead of HCD, which only appeared as a reference paper. At the end of step 3, we excluded all irrelevant papers across the databases for the following step.

The included papers are analysed in detail and ranked in order according to what extent they are relevant to HCD and Industry 4.0, with a focus on the manufacturing areas. We classified them into three groups of inclusion: (DR) 24 directly related papers dedicated to HCD in the context of manufacturing; (PR1) six partially related papers studying HCD but in different contexts; (PR2) 47 partially related papers providing useful information related to HCD: design concepts, design methods, supporting technologies, human diversity, ergonomics, economics, manufacturability, and sustainability. Based on our presented objectives, the following section starts by presenting the overall characteristics of the literature, followed by an in-depth review of case studies—emerging trends, design methods, lessons learnt—and opportunities for future research.

## Literature characterization of human-centred design in industry 4.0

This section provides an overall quantitative picture of the included papers: the trend of research interest associated with the most cited papers, the regions and countries where the papers are made, and, importantly, the transdisciplinary and multidimensional approach in HCD. Subsequently, the in-depth review of case studies presents the emerging trends among the concepts of HCD and design methods, followed by an affinity analysis that categorizes their research outcomes and limitations.

### Overall characteristics

#### Growth rate of research interest

**Fig. 2:**
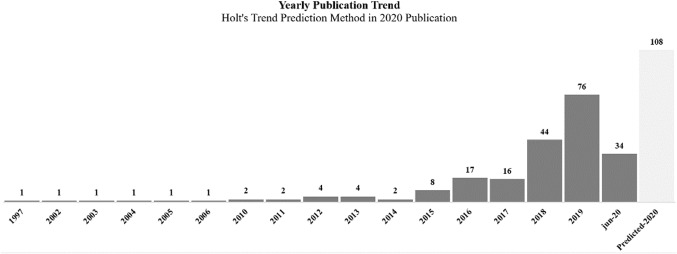
Yearly publication trend with the exclusion of duplicate papers

After excluding the duplicate papers, there are 215 remaining papers whose yearly publication data allow for the extrapolation of two interesting stages from 1997 to the middle of 2020, as portrayed by Fig. 2. First of all, one notices that the topic has gained momentum and research interest in different aspects of HCD. Secondly, for the period 2015–2019, there has been an almost consistent and healthy growth in the number of publications. Obviously, the 2020 data is still incomplete, which shows a lower number of publications than that of the previous years, because this research was carried out in the middle of the current year. Besides, we applied the Hot’s trend prediction method to exponentially conjecture that the research publications could reach 108 papers by the end of 2020. However, the growth rate could be affected due to the global issue of Covid-19.

By examining only 77 included papers, Table 3 presents the most cited papers, accounting for 63% (329 out of 501 total citations). Interestingly, these top-cited papers have almost been published in recent years. This fact shows that the development of HCD has not matured yet, while the scholars have made the references to the recently published papers for new findings instead of citing the previous ones that have not been well generalized in the research community.

**Table 3:** Papers by citations by Web of Science, retrieved from 19 July 2020

Author	Year	Paper title	Number of citations
Zheng et al. (2018)	2018	Smart manufacturing systems for Industry 4.0: conceptual framework, scenarios, and future perspectives	94
Pacaux-Lemoine et al. (2017)	2017	Designing intelligent manufacturing systems through human–machine cooperation principles: a human-centred approach	42
Brenner et al. (2014)	2014	User, use & utility research	30
Fernandez-Carames and Fraga-Lamas (2018)	2018	A review of human-centred IoT-connected smart labels for the industry 4.0	28
Lee and Abuali (2011)	2011	Innovative Product Advanced Service Systems (I-PASS): methodology, tools, and applications for dominant service design	27
Varshney and Alemzadeh (2017)	2017	On the safety of machine learning: cyber-physical systems, decision sciences, and data products	22
Streitz (2019)	2019	Beyond ‘smart-only’ cities: redefining the ‘smart-everything’ paradigm	15
Zhu et al. (2015)	2015	A product-service system using requirement analysis and knowledge management technologies	15
Mourtzis et al. (2018)	2018	Product-service system (PSS) complexity metrics within mass customization and Industry 4.0 environment	14
Leng and Jiang (2017)	2016	Granular computing–based development of service process reference models in social manufacturing contexts	14
Qin et al. (2016)	2016	Exploring barriers and opportunities in adopting crowdsourcing-based new product development in manufacturing SMEs	14
Mazali (2018)	2018	From Industry 4.0 to society 4.0, there and back	14

The top cited paper of Zheng et al. (2018) outlines future perspectives of smart manufacturing systems in which user experience is considered as one of development challenges, and transdisciplinary research is called for future research. Beyond the technical perspectives, the scholars also drew attention to social aspects. Specifically, the work of Mazali (2018) explicitly concluded that one of the key issues for the future is to design a balance between the worker being able to control the process by using their own intelligence and the automation of digital algorithms. This perspective is also agreed upon by the work of Streitz (2019), who graded the equal importance among humans and technologies in ambient intelligence to achieve the smart paradigm.

#### Publication origin

**Fig. 3:**
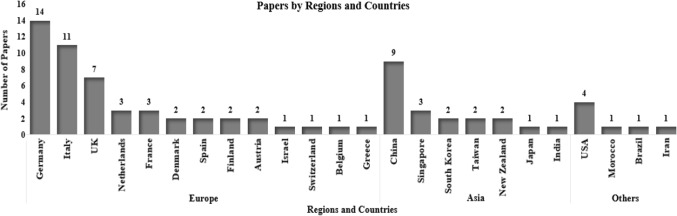
Papers by regions and countries

By taking a detailed look at 77 included papers, Fig. 3 shows that the most influential countries are accounted for by Germany (18%), followed by Italy (14%), and China (12%). In the regions, European countries have shown strong contributions in the field with 65% publications, which was reflected by several pieces of research—*Factories of Future* (European Commission, 2013) and *Platforms for CPSs* (Thompson et al., 2018)—whose recommendation for future research indicates that it has been a long road reaching the systems of *HioTL* at the matured level together with other emerging technologies. Some specific research programs and priorities in the next three decades are extracted as below:

- Human-oriented interfaces for workers: process-oriented simulation and visualization.
- Products and work for different types of skilled and aged labour, education and training with IT support.
- Regional balance: work conditions in line with the way of life, flexible time-and-wage systems.
- Knowledge development, management and capitalisation.

#### Transdisciplinary approach

By examining the journals by which the included papers were published, the transdisciplinary approach of HCD is strongly evidenced by the fact that there are no journals significantly overwhelming other journals. Table 4 reveals two interesting facts. First, the top 11 journals out of 54 journals—which publish 77 included papers—range from varying research disciplines: engineering; computer science; business management; social and philosophy, which is specialized by the journals *Cognition, Technology & Work* and *AI & SOCIETY*. This transdisciplinarity integrates cross-disciplinary perspectives—philosophy, engineering, computer, business, and social sciences—in the context of HCD and transcends their traditional boundaries. This fact addresses the interest in extending the research boundaries of various dimensions of HCD: human diversity, physical to cognitive ergonomics, economics, manufacturability, and social and human-related sustainability.

**Table 4:** Papers by journals

Journal Title	No of Papers	Category	JRC Impact factor	JRC Rank	SJR indicator	SJR rank
International Journal of Advanced Manufacturing Technology	8	Computer Science Engineering	2.633	Q3	0.999	Q1
Chinese Journal of Mechanical Engineering	6	Engineering Mechanical	1.824	Q3	0.531	Q2
Cognition, Technology & Work	3	Computer Science Philosophy Human–Computer Integration (HCI)	1.206	–	0.436	Q3
Business & Information Systems Engineering	3	Computer Science Information Systems	5.873	Q1	1.306	Q1
Journal of Manufacturing Technology Management	2	Engineering & Management	3.385	Q2	1.173	Q1
Journal of Intelligent Manufacturing	2	Computer Science Engineering	4.311	Q1	1.213	Q1
Journal of Ambient Intelligence and Humanized Computing	2	Computer Science	4.594	Q1	0.544	Q1
International Journal of Computer Integrated Manufacturing	2	Computer Science Engineering	2.861	Q2	0.658	Q2
Electronic Markets	2	Business & Management	2.891	Q2	1.006	Q2
Computers & Industrial Engineering	2	Computer Science Engineering	4.135	Q1	1.469	Q1
AI & Society	2	AI & Philosophy HCI	–	–	0.294	Q3

**Fig. 4:**
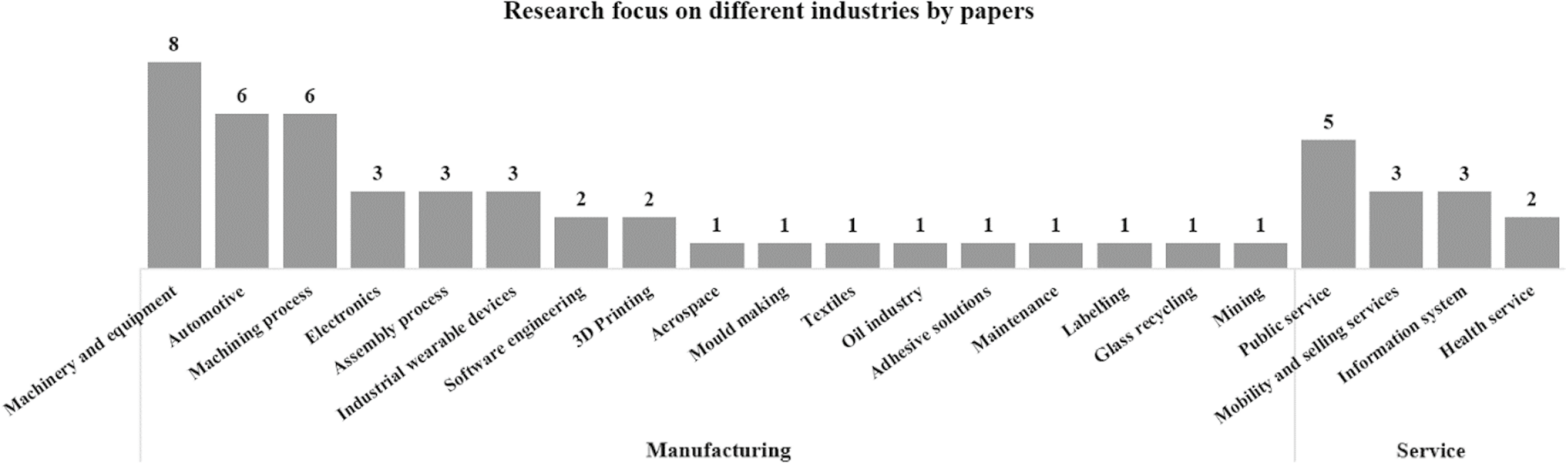
Research focus on different industries by papers

This transdisciplinary approach has also brought different studies across various research contexts, as can be seen in Fig. 4. There are 42 papers out of 77 included papers that clearly indicate their research focuses on specific manufacturing processes and industries: *machinery and equipment* as the top one, followed by *automotive industry* and *machining process*. The adaption of HCD has progressed in more specific fields: *adhesive solutions* was considered as the case study on which Lee and Abuali (2011) tested their methodology of innovative and advanced PSS; smart *labelling* design was developed from the foundation of Industry 4.0 human-centred smart label applications proposed by Fernandez-Carames and Fraga-Lamas (2018); design for *textiles* was implanted with interactive technologies to experiment and enhance fashion emotional design by Wang et al. (2018).

On the other hand, there are 13 papers out of 77 included papers that explicitly adapt HCD in services, for example *public service* for smart housing services—which seamlessly connect humans and machines—by design for HMI with the application of Bluetooth ubiquitous networks (Diegel et al., 2004) or a 3D-based meta-user interface (Mostafazadeh Davani et al., 2018). For the *healthcare sector*, Haber and Fargnoli (2019) emphasized the understanding of human needs and proposed the approach of PSS—the integration of products (hemodialysis devices) and services (e.g., technical support, response time)—for the offering’s value. In the same sector, Gervasi et al. (2020) proposed an evaluation framework—which expresses the perspectives of engineering, cognitive, and social science—of HRC to assess the support of robots for elderly people to reach a specific place.

#### Multidimensional approach

The research methodology is also diverse in both conceptual and empirical research, as evidenced by Table 5. Fifty-six out of 77 included papers (around 73%) take an empirical approach, while the remaining 21 papers (around 27%) contribute to the conceptual findings. Empirical research uses scientific data or case studies for explorative, descriptive, explanatory, or measurable findings, while conceptual research focuses on abstract ideas, concepts, and theories built on literature reviews (Marczyk et al., 2005; Williams, 2011). Those conceptual papers are further categorized into SLR, accounting for four papers (around 5%) that differentiate from traditional narrative review papers (around 22%). The strong point of SLR is a replicable, scientific, and transparent process minimizing bias through exhaustive literature searches of studies and simultaneously providing the traceability of results (Boell & Cecez-Kecmanovic, 2015; Tranfield et al., 2003). Of the 56 empirical articles, 37 papers (around 66%) are qualitative studies and 19 articles (around 34%) are quantitative studies. Those figures explain the current research effort that focuses on describing, explaining, and interpreting HCD is overtaking the research effort on quantification and statistical treatment for supporting or refuting research findings. This fact is reflected by the nature of the social phenomenon being investigated from the human point of view, leading to the difficulty in the generalization of results (Mennell, 1990; Walsh et al., 2015).

**Table 5:** Methodological approaches of included papers

Level of analysis	Conceptual		Empirical		Total included papers
	Systematic literature review	Traditional literature review	Qualitative	Quantitative	
Society level		3	2		5
Company level	2	8	17	5	32
Workstation level			5	2	7
Product level	2	5	14	12	33
Total	4	16	38	19	77

Table 5 also reveals the multidimensional approach of levels of research analysis that range from the level of the product to the levels of the workstation, the company and, finally, society. The research on the level of society and the workstation is still modest in comparison with that of the company or the product, accounting for 12 papers out of 77 included papers (around 16%). The figures show there is reasonable space for further research that deals with HCD at cross-layer levels other than the company and product level, which is also consistent with the future research agenda proposed by the European Commission (2013).

In a broader sense, by applying the qualitative research methodology, Fosch-Villaronga et al. (2020) took a step beyond the company level to gather expert opinions addressing social challenges—ethical and legal issues, job availability—due to the use of social robots. They investigated the challenges from both user perspectives—privacy, autonomy, the dehumanization of interactions—and worker perspectives, such as the possible replacement of jobs by robots. Based on the companies’ perspectives with regard to addressing this level of social concerns with the qualitative approach, Mazali (2018) conducted 40 in-depth interviews with managers of 20 manufacturing companies to accommodate the social needs and organizational contexts that involve multiple stakeholders and new roles of intelligent systems in workflows. In the lower area, the company level is addressed by the business cases and processes. For instance, the work of Hammer et al. (2018) shows an extension of existing business models for *quality of experience* that incorporate user needs and motivation as aspects of the individual dimension. Subsequently, the workstation level concerns the design for human-oriented workstations, for instance, addressed by Gualtieri et al. (2020) who concluded the need to perform an accurate ergonomic assessment at the first phase of workstation design. The last layer of analysis is the product level, whose design object is an artefact or a service solution.

In addition to the transdisciplinary approach—an integration of cross-disciplinary perspectives—in HCD, this multidimensional approach is also evidenced by the cross-layer level—the product and/or service, workstation, company to social level—in which humans are centred.

### In-depth review of case studies

There are 43 papers that report case studies out of 77 included papers (around 56%), as detailed by “the Appendix” (Table 11), which provides a useful source for researchers to make references to design for case studies. Those case studies report the design problems associated with the contexts, data collection, and analysis in both quantitative and qualitative approaches. The review objective is to make contributions to the future research agenda by harmonizing the lessons learnt that reveal the research results and limitations of the case studies. In addition, the subsequent section provides the emerging trend of concepts regarding HCD, followed by the structured harmonization of design methods.

#### Emerging trend

The strategy to categorize the case studies follows the design concepts embraced by the corresponding paper. Those concepts are not always explicitly indicated by the papers that may use the term “human” or “user” and even consider them interchangeable terms. This confusion is also reported by Holeman and Kane (2020) and Bazzano et al. (2017). Therefore, Table 6 structures the description of the concepts associated with their common context of use.

**Table 6:** Variants of HCD in various contexts of Industry 4.0

Design concepts	Description	Context	Authors
Human-centred design (HCD)	Applies physical, cognitive, and social factors to design*—*tools, tasks, machines, systems, and environments*—*for enhancing effectiveness and efficiency: human use and safe	General	Fernandez-Carames and Fraga-Lamas (2018), Grandi et al. (2020), Rossi and Di Nicolantonio (2020)
Human-in/on-the-loop (HioTL)	Indicates a human-in-the-loop system that requires human intervention in maintaining its continual operations; a human-on-the-loop system that shifts the human role to govern its autonomous operations	Cyber physical systems	Zhang et al. (2017), Nahavandi (2017), Kong et al. (2019), Vanderhaegen, (2019)
Human–robot collaboration (HRC)	Studies a collaborative system that achieves common goals shared by humans and robots working autonomously together to perform their assigned tasks through collaboration: knowledge sharing and social negotiations	Cyber physical systems	Richert et al. (2018), Weichhart et al. (2019), Cohen et al. (2019), Fosch-Villaronga et al. (2020), Gervasi et al. (2020)
Human–machine interface (HMI)	Defines a system’s interfaces that allow humans to understand and operate it. This design embraces transdisciplinary knowledge and skills: human factors, industrial design, information processing, and cognitive psychology	Cyber physical systems	Oborski (2004), Lepratti (2006), Wu et al. (2016), Chen and Duh (2019), Ong et al. (2020)
User-centred design (UCD)	Studies an iterative design process whose research subject is not only usability but also user experience: emotional and psychological responses to interaction design	Social-technical systems	Wu et al. (2016), Kymäläinen et al. (2017), Lin (2018), Mazali (2018)
Product-service systems (PSS)	Indicates a system integration of products and services that facilitates customer-focused and co-creation models to deliver value propositions towards sustainability through all its life-cycle stages	Servitization	Grieger and Ludwig (2019), Cheah et al. (2019), Haber and Fargnoli (2019), Leoni (2019), Bednar and Welch (2020), Saha et al. (2020)

The variants of HCD reinforce the findings of the transdisciplinary and multidisciplinary approach—physical to cognitive ergonomics, products and/or services to social-technical systems—towards human diversity, ergonomics, economics, manufacturability, and social and human-related sustainability. Based on the understanding, Table 7 captures the emerging trend that provides insights into six concepts summarized in chronological order.

**Table 7:** Emerging trend of HCD concepts across case studies towards Industry 4.0

Design concepts	2005–2007	2011–2013	2014–2016	2017–2020	Total cases
Human-centred design (HCD)	1	1	1	11	14
Product-service systems (PSS)	–	1	1	11	13
User-centred design (UCD)	–	–	1	7	8
Human-in/on-the-loop (HioTL)	–	–	–	3	3
Human–machine interface (HMI)	–	–	2	1	3
Human–robot collaboration (HRC)	–	–	–	2	2
Total cases^a^	1	2	5	35	43

^a^Total cases for each concept summed from “Appendix” (Table 11)

The top three concepts—namely HCD, PSS and UCD—that account for 35 out of 43 case studies (around 81%) are the most frequently and recently used concepts during the last three years. HCD is the most popular term, although it originated somewhere in the 1400s to systematically improve design for procedures and tools to accomplish the work (Nemeth, 2004). HCD has changed dramatically in the context of Industry 4.0, where scholars have expanded the research of physical ergonomics to systems including humans. Specifically, the case studies are designed in various implementation scales in different contexts: the product level by testing the method of *individual product innovation design* in solving bicycle problems based on ergonomic perspectives (Wu et al., 2013); the company level by validating the proposed model of the *artificial self-organizing manufacturing control system* explicitly putting humans in the centre of the system design (Pacaux-Lemoine et al., 2017). Beyond technology, the trend of market personalization has received increasing attention from researchers. The literature witnesses the increasing number of case studies that pertain to the concepts of PSS and UCD. The case studies also distinguish clearly between PSS and UCD by the way that PSS focus on business models at the company level while UCD experiments focus on human experiences about design for product and/or service solutions at the product level in consideration of human diversity and social aspects.

On the other hand, the case studies related to the concepts of HioTL, HMI and HRC are not well accounted for. One of the technical challenges is that the boundaries between technologies and humans are increasingly fuzzy: language processing, social robotics, artificial intelligence, cyber physical systems, virtual reality, and augmented reality. This phenomenon is blurring the limits of where the human ends and technology starts (Frauenberger, 2019; Gervasi et al., 2020; Weichhart et al., 2019; Wojtynek et al., 2019). Moreover, recent research tends to focus on technical aspects instead of tackling existing problems related to error-prone interaction between human and non-human actors (Klumpp et al., 2019; Song et al., 2016).

Another fact shows that the research community has responded in a determined way—35 case studies during the period of 2017–2020, which greatly exceeds other periods—to the call for empirical research in the field (Benabdellah et al., 2019; Kadir et al., 2019). This effort, which is worthy of emphasis, reveals an increasing interest in empirical studies, which brings research and industrial applications closer together. This trend also aligns with the future research recommendations: *Factories of Future* (European Commission, 2013) and *Platforms for CPSs* (Thompson et al., 2018). The following deep analysis manifests the design methods connected with supporting technologies that the papers embrace in order to realize the effort in question.

#### Design methods

**Fig. 5:**
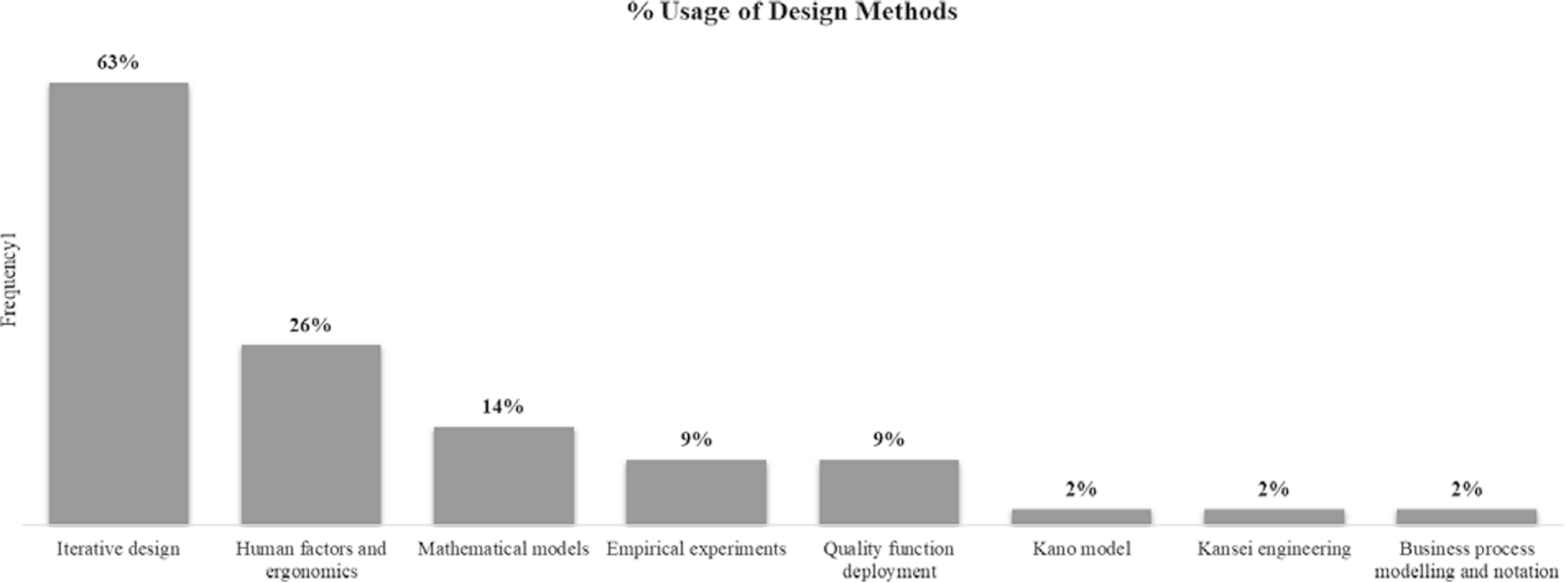
Design methods applied by the reviewed case studies. ^**1**^Frequency divided by the total number of case studies (43 case studies) derived from “Appendix” (Table 11)

Norman (2016) explains that “the human mind is exquisitely tailored to make sense of the world” (p. 2). This ability requires products and/or services that are designed for easy interpretation and understanding. Therefore, methods for design must define procedures, techniques, aids, or tools to discover the minds of humans—users, customers, stakeholders—that serve as key inputs resulting in well-designed solutions. Figure 5 captures the frequency of design methods that are discussed in four generic groups: discovery, clean-up, engineering, and experiment.

Around 63% of case studies make the most of *iterative design*: knowledge obtained through the discovery is assured by an iterative process of idea exploration, gathering, and assessment. This method contains a bundle of procedures, techniques, and tools—participatory design, interviews, questionnaires, focus groups, scenario observation, field studies, prototyping—for searching and matching design ideas with the human mind. These approaches help designers focus on human diversity to gain critical design inputs and feedback: requirements elicitation acquired from maintenance professionals by field studies (Kaasinen et al., 2018), human perception of different stakeholders by focus groups (Turetken et al., 2019) and usage difficulties of non-expert users by scenario observation (Song et al., 2016). On the basis of questionnaires, Kong et al. (2019) also studied and called user frustration “the key pain spot” in the context of industrial wearable systems. They also pointed out countermeasures—confinable and reconfigurable modularized hardware sets—that address the usage, cognitional, and operational issues, and reduce the complexity and cost in the design solutions considering various aspects: ergonomics, plug-and-play features, and manufacturability. The modular approach is also comparable to *product platform design* that tackles the issues regarding manufacturability—product customization, variety, and commonality between products—and brings a competitive advantage: reduction in design effort and time-to-market for future generations of products (Farrell & Simpson, 2003; Martin & Ishii, 2002; Simpson, 2004). This is further evidence to show the necessity of the transdisciplinary and multidimensional approach within which an engineering method can also be applicable in the context of HCD to integrate human and non-human factors: human diversity, ergonomics, economics, manufacturability, and sustainability.

In addition to the acquisition of human needs and requirements, iterative design is also suitable for investigating “what-if” scenarios on design solutions. For instance, Kymäläinen et al. (2017) and Harwood et al. (2019) built fiction prototyping—video-illustrated and tangible interaction tools—to facilitate human-centred perception and cognition of the future potentials of products and/or services. This so-called design fiction—an interactive and tangible approach—evaluates alternative design solutions or criticizes existing ones (Knutz et al., 2014) before they are manufactured and/or delivered to customers, which enhances the robustness of iterative design by deeply understanding human experience.

Even though an effective understanding of human requirements is vital for well-designed solutions, this task is difficult due to various subjective human ideas: prioritization, complexity, imprecision, and vagueness. Clean-up is significantly more challenging for requirements of services than those of products (Haber & Fargnoli, 2019; Song & Sakao, 2016). To respond to the challenge, 6 out of 43 case studies (14%) deal with fuzzy inputs and multiple-criteria decision making by applying *mathematical models*: analytic network process (ANP), Thurstone’s Law of Comparative Judgments (LCJ), fuzzy set theory, and geometric vectors. While Zhu et al. (2015) took advantage of ANP to determine and prioritize the importance weights of engineering characteristics derived from a set of different customer requirements, Haber and Fargnoli (2019) prioritized customer requirements by the LCJ that transforms the customer preferences into scale values and then represents the importance of each preference. To quantify the complexity, Mourtzis et al. (2018) proposed a 2D geometric vector to estimate the product and service’s design complexity, which is defined by information content, quantification of information, and diversity of information. This quantification of complexity supports the decision-making process on alternative design solutions, taking manufacturability into account. To deal with imprecision and vagueness, Chen et al. (2016) evaluated the users’ perceptual images and feelings about products by the use of the fuzzy membership degree of emotional semantic descriptive words (e.g. traditional-modern, geometrical-organic, romantic-realistic). They also used a statistical method—principal component analysis—to cluster the varying user perceptions and feelings into homogeneous groups of design characteristics. Similarly, Leng and Jiang (2017) clustered similar individual service design processes into homogeneous bundles of services by applying a granular computing method—fuzzy set theory combined with quotient space theory for classification (or clustering) of uncertain complex problem (Zhang & Zhang, 2010). Taking both customer and engineering subjective ideas, Chen (2016) carried out the fuzzy analytic hierarchy process (AHP) to develop good quality design based on the imprecise relationship between engineering experience (robust design, design optimization, design cognition) and customer experience (requirements management, ergonomics design). Based on that, the author also proposed a linear programming model to optimize the total profit of the product mix-experience portfolio, taking economic considerations into account. This cost–benefit analysis needs to be embraced because its importance is stated by several authors, especially with regard to the entire life-cycle cost analysis (Anke, 2019; Heidari et al., 2020; Rodriguez et al., 2020). These mathematical methods are useful in dealing with the multiple-criteria decision making and fuzziness (uncertainty) under their own assumptions, constraints, and computing capability, requiring practitioners to be transdisciplinary and understand properly the methods in their context of use. For references regarding these methods, refer to the work of Golden et al. (1989), Kubler et al. (2016), and Liu et al. (2020).

In addition to the discover and clean-up, 26% of the case studies apply *human factors and ergonomics* to understand and evaluate quantitatively the interactions—physical and cognitive ergonomics—among humans and other actors (e.g., design artefacts, virtual objects, system interfaces, industrial workstations) from the engineering perspective. This method is not only for the expected cost saving, but also for the higher process efficiency that can be realized by shedding light on human factors and incorporating human needs and behaviour in a healthy, safe, efficient and enjoyable manner (Labuttis, 2015; Soares & Rebelo, 2016). In the context of Industry 4.0, this method is also supported by the digital technologies—virtual and mixed reality, eye-tracking systems, digital modelling and simulation for virtual workplaces—to facilitate designers to capture and analyse design data that span from the physical to cognitive level. On the cognitive level, Wu et al. (2016) studied the relationship between interface complexity and user diversity—novice and expert (human background)—by measuring users’ psycho-physiological data (eye-movement research) combined with questionnaire evaluation methods: NASA-task load index and Questionnaire for User Interface Satisfaction (QUIS) to measure operators’ subjective feelings and workload throughout the experiment. These eye-movement data provide insights into the visual, cognitive, and attentional aspects of human performance (Duchowski, 2002). In addition to the psycho-physiological analysis, Richert et al. (2018) surveyed participants’ personality dimensions—agreeableness, conscientiousness, neuroticism, openness to experience—to measure the performance and human perception of hybrid human–robot collaboration. On the physical level, Caputo et al. (2019) carried out an appraisal for the human-centred workplace design by reproducing a virtual workplace in which digital human modelling simulates the whole human task towards preventive ergonomics. Peruzzini et al. (2019) also designed the virtual workstation with preventive ergonomics by the use of digital technologies: virtual and mixed reality. They also used questionnaire methods to quantitatively measure postural comfort: Rapid Upper Limb Assessment (RULA) and Ovako Working Posture Analysis System (OWAS). The case studies apply a wide range of assessment methods regarding human factors and ergonomics: from simple checklists to more complex techniques; from physical ergonomics—for human use and performance (e.g., musculoskeletal symptoms, body posture, low back disorders)—to cognitive ergonomics—for human perception and cognition (e.g., mental stress, emotional stress, situation awareness). In addition, the work of Tillman et al. (2016), Forsythe et al. (2017) and Dalle Mura and Dini (2019) provides a good source of numerous methods for human factors and ergonomics that allow for achieving the various objectives of both manufacturability and social sustainability.

To bridge the gap between human requirements and engineering characteristics, four out of the 43 case studies apply *quality function deployment* (QFD), which originated in the automotive industry and has been being used with different applications in diverse fields for five decades (Kowalska et al., 2018; Zairi & Youssef, 1995). This method identifies human-centred requirements, classifies the importance of those requirements, defines engineering characteristics that may meet those requirements, allows for verification of design conflicts among them, and then prioritizes design solutions. In the analysed case studies, this method is also integrated with different methods—application space map and innovation matrix (Lee & Abuali, 2011), ANP (Zhu et al., 2015); AHP, fuzzy AHP, entropy weight method (Ma et al., 2017); LCJ and Kano model (Haber & Fargnoli, 2019)—to enrich the prioritization and segmentation of the design requirements. The requirements after the cleanup are further converted into the engineering parameters by the QFD. For further reading, the work of Chan and Wu (2002) and Prasad (1998) may be of interest to the reader.

Furthermore, other methods also include the *Kano model*, *Kansei engineering*, *business process modelling, and notation* (BPMN). While Haber and Fargnoli (2019) applied the Kano model to prioritize and classify customer requirements into four different categories—must-be, one-dimensional, attractive, indifferent—for the segmentation of customer value propositions, Wang et al. (2018) parametrically linked the customer's emotional responses—physical and psychological—to the properties and characteristics of a product and/or service. If these methods focus on a particular process in design (requirement elicitation converted into engineering characteristics), Prinz et al. (2019) highlighted the use of BPMN to represent workflows—a graphical modelling language for all kinds of business processes. The BPMN is useful for examining a graphical description of design processes to different levels of granularity and discovering inconsistencies and/or differences in sequential steps, conflicting names, or acronyms, to name a few. Even though the methods have only been mentioned one time by the 43 case studies, they have been adapted and applied by different fields for years. Several publications are interesting works that may help readers have a better idea about the Kano model published by Zhao et al. (2020) and Shahin et al. (2013), Kansei engineering reviewed by Shiizuka and Hashizume (2011) and Coronado et al. (2020), BPMN studied by Ko et al. (2009) and Chinosi and Trombetta (2012).

Lastly, another way of gaining knowledge in design is *empirical experiments*, which account for four out of the 43 case studies. This method is useful for understanding what-if scenarios by different design configurations: an assisted versus collaborative robotic system that supports workers in a plug-and-produce workstation (Wojtynek et al., 2019), an automatic speed versus adaptive cruise control system for pedagogical learning supports (Vanderhaegen, 2019), delivery of health care services for seniors between a community hospital and social service agency (Hoe, 2019), augmented reality that supports trainers versus trainees in phone repairing operations (van Lopik et al., 2020). Those empirical experiments allow for designing hypotheses and gaining knowledge by means of direct and indirect experience. However, this method requires knowledge of the experimental setup and validation; it also has limited generalization of results due to controlled settings (Kulyk et al., 2007).

In summary, the case studies apply various methods that are categorized in the four generic groups—discovery, clean-up, engineering, experiment—associated with supporting technologies to tackle different problems, which requires the transdisciplinary approach for understanding and applying the methods in their proper context of use. While iterative design is power in discovering the human mind (needs, perception, cognition), mathematical models prioritize and classify those human inputs and support the decision-making process on design alternatives. Furthermore, human factors and ergonomics enrich the understanding of interactions—physical to cognitive ergonomics—among human and non-human actors with the support of digital technologies: virtual and mixed reality, eye-tracking systems, digital modelling and simulation for virtual workplaces. To convert the voice of humans into engineering parameters, the case studies have diverse approaches—QFD, Kano model, Kansei engineering, BPMN—and are used in different combinations. Finally, the empirical experiments gain knowledge based on the investigation of what-if scenarios under the human perspective, which is useful for iteratively improving and testing design solutions. Besides, researchers and practitioners alike also benefit from other relevant engineering methods—product platform design (Simpson et al., 2014), design for manufacturability and concurrent manufacturing (Anderson, 2014), to name a few—that embrace the transdisciplinary and multidimensional approach to deal with a multi-objective design problem towards human diversity, ergonomics, economics, manufacturability, and sustainability.

These various methods dealing with different problems in diverse contexts of use lead to different lessons learnt in the form of their research results and limitations. The following lessons learnt are useful for subsequent researchers to choose proper research areas and advance research contributions to the field by avoiding the research limitations.

#### Lessons learnt

One way to organize the case studies sharing mutual facts and document them as the lessons learnt is to use an *affinity analysis,* which is also known as the *KJ method* and applied in various fields (Awasthi & Chauhan, 2012). The information captured during the analysis is tabulated by “Appendix” (Table 11), providing researchers useful details about design for case studies. Based on the analysis output, Table 8 categorizes the case studies’ results and limitations into six groups of research results (RR) and four groups of result limitations (RL).

**Table 8:** Results and limitations of research case studies in literature

%^a^	RR codes	RR description	RL description	RL codes	%^a^
47	RR2	Explored design success factors	Limited statistical power in result validation	RL1	60
23	RR1	Achieved engineering objectives of design	Lack of generalizability of results	RL2	56
23	RR6	Provided supporting design frameworks	Require supporting methods to facilitate the implementation of proposed models	RL4	30
12	RR3	Validated the effect of human diversity	Lack of validation on effectiveness of the proposed solutions	RL3	23
9	RR5	Provided transdisciplinary frameworks			
7	RR4	Visualized design scenarios			

^a^Frequency divided by the total number of case studies (43 case studies) derived from “Appendix” (Table 11)

**Fig. 6:**
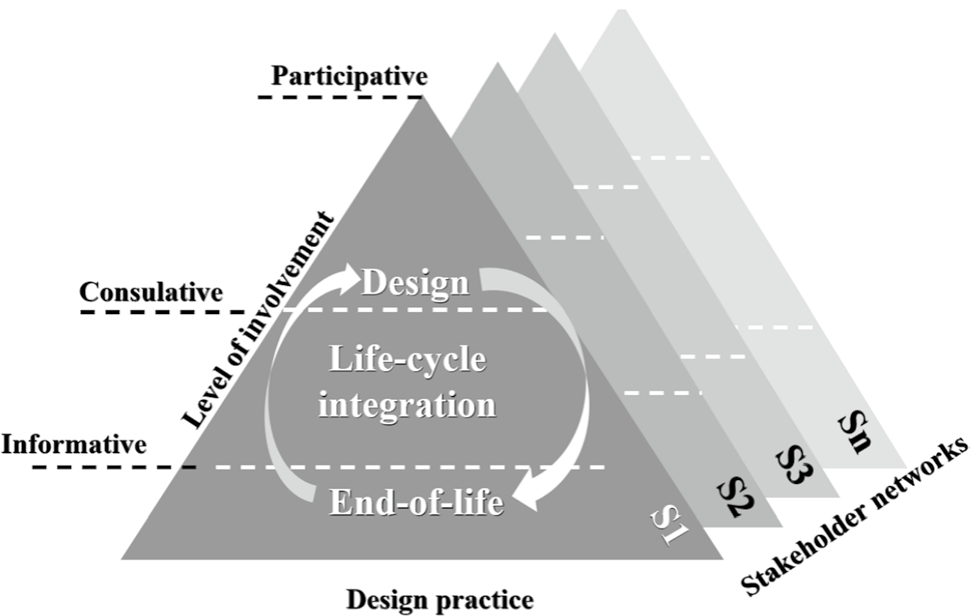
A triangular decision-making diagram in HCD, encompassing design decisions on who in the stakeholder networks (S1, S2, S3, Sn) will be involved, at what levels of involvement, where the involvement will take place in each through-life phase, and what design knowledge should be exploited within the scale of physical to cogitive ergonomics

One of the most attractive outcomes those case studies reported is the exploration of the design success factors—which are denoted as RR2 accounting for around 47% of the case studies—revealing how the successful deployment of design oriented to humans can be generalized in various contexts. Figure 6 structures those success factors as a triangular decision-making diagram:

- *Stakeholder networks*: the organizational, social, and environmental contexts—which involve stakeholders (e.g., users, customers, employees, suppliers, distributors, partners, regulators, etc.) through the life-cycle design process—are essential for enhancing the credibility of information and promoting the sharing of transdisciplinary knowledge as valuable design inputs (Chen, 2016; Mazali, 2018; Schulze et al., 2005; Witschel et al., 2019). The diversity in interests and expectations of the stakeholders needs to be respected and analysed to comprehend the impact of stakeholder interactions and their features at different life-cycle design phases: design, production, delivery, service, maintenance and end-of-life cycle (Mourtzis et al., 2018; Turetken et al., 2019; Zhang et al., 2020). In this respect, the involvement of the users or customers in the early development stage is well realized (Chen et al., 2016; Grieger & Ludwig, 2019; Hoe, 2019).
- *Levels of involvement*: the engagement modes of stakeholders are depicted by three levels of involvement. These levels include the informative level in which stakeholders only provide and receive design information; the consultative level in which they comment on pre-defined design scenarios; and the participative level in which they make influencing decisions on a design process, which is a higher level of engagement than that of the informative level, which only considers stakeholders as information sources in the design process (Schulze et al., 2005; van Lopik et al., 2020).
- *Design practice*: the design development—which responds to the extents to which the data about users, customers, and other relevant stakeholders should be properly obtained and analysed—needs to be defined. These data include physical activities, behaviours, opinions, feelings, personalities, and physiological responses (Lin, 2018; Peruzzini et al., 2019; Richert et al., 2018; Wang et al., 2018). They are explicitly classified into two groups: physical ergonomics—which emphasizes physical characteristics—and cognitive ergonomics, which reflects the integration of cognition thinking and cultural characteristics—individual aesthetic habits, national, ethnic cultural differences—to address social-technical aspects in the context of Industry 4.0 (Bednar & Welch, 2020; Fosch-Villaronga et al., 2020; Zhou et al., 2012).

The knowledge management of these design data is well expressed as an enabling success factor that can be exploited by digital technologies. These technologies facilitate the collection, organization, retrieval, and reuse of design knowledge in an effective manner. While Fu et al. (2019) took advantage of IoT solutions (sensors) for user data collection—unintentional behaviour, emotion, culture—and artificial intelligence for data processing, Vanderhaegen (2019) and Grandi et al. (2020) made use of digital and mixed reality simulation in measuring human factors—physical stress, physiological data—and evaluating their design experiments. Instead of starting from scratch, Zhu et al. (2015) and Leng and Jiang (2017) established mathematically a collection of semantic commonalities derived from historical design ontology-based databases—activities, functions, concepts, process sequences—to build a knowledge platform from which a stream of new derivative products and services can be efficiently developed. The objective is to design for variety and custom solutions, enabling designers to not only save time and cost but also make the most of the experience and expertise that were dedicated to the past design activities. The method used to build the knowledge platform is also comparable with product platform design, which has been maturely researched over the last decade (Simpson et al., 2006, 2014) and is a useful source regarding methods and applications for researchers in the field of product and/or service design.

The second group is the engineering objectives of design (RR1) that are converted into key performance indicators to quantify the effectiveness of the proposed models or frameworks. Around 23% of the case studies indicate that their proposed solutions achieve the engineering objectives: avoidance of ergonomic risks (Caputo et al., 2019; Ceccacci et al., 2019), improvement of productivity and simultaneously biomechanical workloads (Gualtieri et al., 2020; Wojtynek et al., 2019), production performance in terms of quality and engineering time (Pacaux-Lemoine et al., 2017; Prinz et al., 2019). Furthermore, Wu et al. (2013) proposed a multi-function and modular method for design focusing on human anthropometrics—the branch of ergonomics that deals with measurements of the physical characteristics of human beings (Pheasant, 1990)—and extending products’ service life towards sustainability. Similarity, Chen et al. (2016) applied a clustering method for product family design based on anthropology—research in understanding human culture, society, and difference (Monaghan & Just, 2000)—to improve the agility of the design process towards manufacturability. This product family design allows designers to not only utilize existing design methods from the product platform to form a series of products, but also gain inspiration from different ethnic groups—human diversity with distinct cultural traits—to extract ideal design elements. In another aspect, Chen (2016) emphasized directly the cost–benefit analysis of design quality, taking into account two economic elements: estimated profit; total cost comprising R&D cost, market capital, and design quality for market share. The reported figures prove the robustness and performance of a system—human diversity, ergonomics, economics, manufacturability, sustainability—can be achievable with the approaches of HCD.

The next research interest is to provide supporting design frameworks (RR6) that facilitate the design process by providing systematic thinking—the use of the integrated novel design methods (innovation matrix, application space mapping, QFD) and Lean initiatives (avoidance of valueless reworks and activities)—towards economic sustainability (Lee & Abuali, 2011; Pezzotta et al., 2018). Other studies focus on design solutions for complexity and uncertainty: incomplete information regarding human requirements (Haber & Fargnoli, 2019); the changes in human preferences (Lin, 2018); decision making on different design alternatives for mass customization towards manufacturability (Mourtzis et al., 2018); interaction requirements among non-human—smart manufacturing devices/tools, core enterprise business systems (ERP, SAP)—and human actors (manufacturers, designers, users) (Mostafazadeh Davani et al., 2018; Song et al., 2016; Zhang et al., 2020); adaptation of design processes to the context of small-and medium-sized enterprises (Adrodegari & Saccani, 2020; van Lopik et al., 2020). These studies tackle different problems scattered across life-cycle design phases, useful to consider in relation to further research to address the relevant problems in a comprehensive way.

Around 12% of the case studies made an effort to validate the effect of human diversity on the design outcomes (RR3). They concluded with the important inclusions of individual differences—background, age, gender, education, cultural influences, privacy management—in design. Statistically, Wu et al. (2016) confirmed that information overload in interface design increased cognitive workload for novice operators compared to expert operators and therefore decreased user efficiency. Similarly, Van Acker et al. (2020) concluded statistically that higher acceptability of wearable mental workload monitoring was associated with being a woman (for trust in the technology), higher technology readiness—the willingness to accept new technologies and security about private data (Victorino et al., 2009)—and lower educational backgrounds. Besides, lack of considerations regarding specific classes of difference between humans leads to major effects on design outcomes in various design contexts: age with older people (aged 55–75 years) in safe driving (Jung et al., 2017) and health sector (Hoe, 2019); cultural influences (Russians, a Frenchman, a Chinese) in the experiment of long-term isolation in a limited room space (Boy, 2018). These studies address the concern that if design does appreciate individual differences towards the multidimensional approach—considering not only product and/or service design but also social aspects—this could avoid the thwarting of all research efforts and the subsequent lessening of potential benefits.

In addition to the multidimensional approach, four studies also directly address the need for collaborative design frameworks (RR5): the transdisciplinary approach during the life-cycle design phases. Ma et al. (2017) exploited common expertise of transdisciplinary teams to convert customer requirements into semantic requirement groups that were subsequently transferred into product design specifications through the use of QFD. Based on the perspective of cross-cutting collaboration for advanced business intelligence, Kong et al. (2019) structured a common platform design of wearable-enabled applications with three aspects of manufacturability: re-configurability, robust architecture, and design scalability. This platform allows standardization by taking advantage of plug-and-play features and modular approaches to integrate human and non-human actors: artificial intelligence, virtual reality, IoT, cloud computing, and cloud-based cyber systems (enterprise resource planning, manufacturing execution systems, warehouse management systems). In addition to manufacturability, Anke (2019) and Turetken et al. (2019) addressed directly the aspects of life-cycle cost analysis in the context of smart services. Specifically, Anke (2019) assessed the profitability of a smart service at an early stage of service design by developing a web-based tool prototype by which project teams from different disciplines collaborate in the design and evaluation process. In a broader sense, Turetken et al. (2019) promoted the transdisciplinary and iterative approach in which a network of actors—providers, customers, authorities, retailers, event organizers—co-creates the value-in-use for customers and generates benefits—financial and non-financial characters—for all network partners moving towards sustainability. Each study focuses on an important aspect of design—human diversity, ergonomics, economics, manufacturability, sustainability—that needs to be considered together in a transdisciplinary and multidimensional approach for future research.

In the last group of research interest, three studies present experience-driven approaches that visualize design scenarios (RR4) regarding future possibilities to exploit human experience. Based on design fiction, both Kymäläinen et al. (2017) and Harwood et al. (2019) demonstrated the usefulness of the video-illustrated prototype in avoiding the difficulty of interpreting abstract verbal descriptions of new design. This method enables designers to interactively envisage a spectrum of “what if” scenarios towards human experience that may then be explored by using the range of other design methods: focus groups, interviews, and questionnaires. Besides, Kaasinen et al. (2018) made the most of the technologies in Industry 4.0—wearable technologies, virtual and augmented reality—to visualize the human experience of future maintenance work: feeling competent, feeling connected to the work community, feeling a sense of success and achievement by performing better in jobs. These studies go beyond technical design towards the multidimensional approach: they go from the technical to the social aspects.

Even though all case studies reported positive outcomes, four groups of result limitations are also acknowledged. The most frequently reported limitation is the lack of statistical power in result validation (RL1)—accounting for 60% of total analysed case studies—and the rest is undefined due to limited information for making the conclusion. The lack of statistical power shows limitations in experimental set-up conditions: low sample sizes, lack of fitting in target participants, lack of sound statistical studies, and other biased experimental aspects (Pacaux-Lemoine et al., 2017; Richert et al., 2018; van Lopik et al., 2020). This limitation is followed by the lack of generalizability (RL2) showing the insufficient evidence of the extent to which findings from one study in one context can be applied and reproduced to other contexts. Specifically, 56% of the case studies are constrained and required to be tested by further quantitative methods to prove the transferability of their observed results to other usage contexts (Adrodegari & Saccani, 2020; Haber & Fargnoli, 2019; Kong et al., 2019; Witschel et al., 2019). The next limitation is categorized as incomplete solutions to implement the proposed models (RL4)—accounting for around 30% of the case studies—claiming the quality of the proposed models will depend on other external factors. These factors include the “manual” processing of the proposed models, resulting in application difficulties (Ceccacci et al., 2019; Zhang et al., 2020), which requires additional efforts in further development of supplementary methods and applications to achieve model completion in real contexts (Grieger & Ludwig, 2019; Leng & Jiang, 2017; Lin, 2018; Peruzzini et al., 2019). Finally, around 23% of the case studies do not explicitly provide the validation of effectiveness of the proposed solutions (RL3), which emphasizes the need for future research for their validation in various contexts of usage; otherwise, the practical effectiveness of the proposed solutions from the studies is limited (Ceccacci et al., 2019; Haber & Fargnoli, 2019; Witschel et al., 2019).

**Fig. 7:**
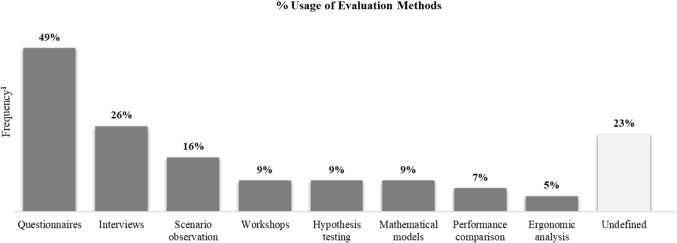
Evaluation methods applied by the case studies reviewed. ^**1**^Frequency divided by the total number of case studies (43 case studies) derived from “Appendix” (Table 11)

These limitations are explained through the evaluation methods—which are different from the design methods used as procedures or processes for attaining research findings—applied by the case studies to validate their corresponding research findings. Figure 7, which is visualized from the detailed data of “Appendix” (Table 11), shows the top four evaluation methods accounted by qualitative methods: questionnaires, interviews, scenario observation, and workshops. These methods validate the effectiveness of the corresponding proposed models by capturing and communicating the participants’ feedback via different means, leading to a potential lack of robustness in research and encompassing subjectivity and bias in research conclusions (Jung et al., 2017; Richert et al., 2018; Van Acker et al., 2020), which is followed by insufficient generalizability, as analysed above.

Although there is a small portion of case studies applying quantitative methods—hypothesis testing and mathematical models (around 9%), performance comparison (around 7%), and ergonomic analysis (5%)—the validation of the case studies’ findings is still questionable. Specifically, by applying the hypothesis testing, L. Wu et al. (2016) made an effort to carry out a case study of eye tracking with 38 participants that compared three levels of interface complexity in LED manufacturing systems, resulting in the statistical conclusion of interface complexity and user background affecting the user experience. However, the study failed to prove sufficient statistical power, showing its proper selection of sample size. Moreover, the sampling procedure included only the participants who were all from the same company, leading to biased results and affecting the generalizability of research outcomes. Out of 43 case studies, Ceccacci et al. (2019) and Gualtieri et al. (2020) conducted ergonomic analysis to validate the effectiveness of their workstation design—productivity, human postural comfort—with a sample size of only two participants. This small sample size, due to its lack of generalizability, requires further research to validate the studies’ applicability in a real context with human diversity. This problem was further evidenced by Van Acker et al. (2020) who reported that, statistically speaking, the replication of their case study’s findings found in the first experiment was not successful in the second experiment carried out within the same research context, so leaving the conclusion inconclusive. These limitations lead to a lack of robustness in research findings and reduce applications of these studies in industry and research alike.

In summary, the research efforts contributing to the realisation of human roles in Industry 4.0 span six groups of research results: exploration of design success factors, achievement of engineering objectives, provision of supporting design frameworks, validation of the effect of human diversity on design, provision of transdisciplinary frameworks, and visualization of design scenarios. Each study focuses partially on its own defined aspects, which provides a useful reference for future research that combines the transdisciplinary and multidimensional approach towards human diversity, ergonomics, economics, manufacturability, and sustainability in a comprehensive way. Besides, it is worth realizing the lessons learnt in order to overcome the acknowledged limitations—limited statistical power in result validation, lack of generalizability of research findings, further requirements for the supporting methods, lack of validation of the effectiveness—and enhance the robustness of the research findings. This will inspire research applications to both industry and research. Finally, the following section discusses the results of the in-depth review and ends with future research opportunities.

## Discussion and opportunities for future research

The analysis of the overall characteristics of the literature regarding HCD reveals its nature and evolution towards Industry 4.0. Various disciplines have made efforts to integrate human roles into the design process, spreading extensively from artefact and service designs to system designs, taking social manufacturing contexts in Industry 4.0 into account. The topic has gained clear momentum, and interest in different concepts of HCD has increased exponentially. This phenomenon leads to evidence of evolution in HCD, whose characteristics and contextual variants—HCD, PSS, UCD, HMI, HioTL, HRC—have evolved in different disciplines across the value chain to tackle new requirements of Industry 4.0. Specifically, HCD is not only applied for the design of procedures or tools to accomplish a task but is also required to have a transdisciplinary approach. This approach ranges from physical ergonomics—for effective and safe human use—to cognitive ergonomics—for treating personality styles. Another piece of evidence is the multidimensional approach of HCD, whose unit analysis originates from design for the product and/or service level to the workstation and company level, and extends to the level of society: ethical, legal and social concerns have risen along with Industry 4.0. However, concerning the industrial state of the art in this topic, there is a lack of evidence of research with full-scale real implementations that go into any detail on cross-level designs that range from the artefact to the social level from which human issues—privacy, ethnic cultural differences, personality styles—are taken into account within transdisciplinary and multidimensional design thinking. Although an increasing number of studies integrate humans in smart manufacturing, many of them limit research scope to physical ergonomics: human factors and ergonomics on operational levels (Kadir et al., 2019; Pacaux-Lemoine et al., 2017; Peruzzini et al., 2019; Wojtynek et al., 2019). Therefore, future research needs to pay attention to the transdisciplinary and multidimensional approach.

Moreover, the changes that trigger Industry 4.0 have impacted throughout the value chain in which the human roles have been shaped in the different phases of the value chain, requiring new approaches to integrate humans in the cycle. This phenomenon also leads to the different variants of HCD as an evolution evidenced by the in-depth review of case studies. Those concepts have been widely studied in recent years, and there is no clear evidence for their maturity, which is further emphasized by the number of conceptual and empirical papers associated with the case studies found in the literature review. In particular, the terms HCD, PSS and UCD have received the most attention in the literature, showing their emerging trend of catching up with the challenges of dynamic environments and diverse changes in the design requirements aimed at personalization and sustainability. To realize the full potential of smart manufacturing, however, the other concepts of HioTL, HMI, and HRC also deserve more attention not only in conceptual research but also in empirical experiments. This is a good indication for both industry and research to pay attention to the numerous research efforts in exploring the various concepts of HCD to tackle the challenging requirements of industry 4.0. In this respect, an interesting consideration for future research would be to try to better unify the relationships between those concepts in order to embed them completely into the cornerstone of Industry 4.0 infrastructure.

In addition, the challenges in Industry 4.0 also call for diverse design methods that tackle different problems across the life-cycle design phases in the transdisciplinary and multidimensional approach. To respond to the call, the in-depth review of case studies captures a wide range of design methods categorized into four generic groups—discovery, clean-up, engineering, and experiment—associated with supporting technologies. While the discovery makes the most of the iterative design—participatory design, interviews, questionnaires, focus groups, scenario observation, field studies, prototyping—to discover human needs and requirements, the clean-up encompasses the mathematical models—ANP, LCJ, fuzzy set theory, geometric vectors—to classify and prioritize the design requirements and make multiple-criteria decisions on design alternatives. Subsequently, the group of engineering methods—human factors and ergonomics, QFD, Kano model, Kansei engineering, BPMN—converts the requirements into engineering characteristics and establishes the design process flow to centre design on humans. Lastly, the case studies carry out the experimental setups for understanding what-if scenarios by different design configurations, which is useful for iteratively improving and testing design solutions from the human perspective. Besides, the support of digital technologies—virtual and mixed reality, eye-tracking systems, digital modelling and simulation for virtual workplaces—enables designers to capture and analyse design data in an efficient way. Due to varying methods in design, it is helpful for researchers and practitioners who are transdisciplinary and understand properly the methods in their context of use. In addition to the design methods, some other engineering methods available in the literature—product design platform (Simpson, 2004), mathematical multi-objective models taking human factors and ergonomics into account (Dalle Mura & Dini, 2019)—are also worthwhile complementing the design toolkit for both products and/or services to acquire multiple design objectives—human diversity, ergonomics, economics, manufacturability, and sustainability—through the transdisciplinary and multidimensional approach in HCD.

Furthermore, the literature review also provides the detailed and useful information extracted from the analysed case studies in the subsection *lessons learnt*, showing the diverse applications of these concepts in different industrial contexts associated with the insights they provide. These lessons learnt to represent various research results associated with limitations that are captured and harmonized in homogeneous groups: six groups of research results and four groups of research limitations. Given the results, the design success factors—which are again reflected by the transdisciplinary and multidimensional characteristics—are the proper design decisions: the stakeholder networks; levels of involvement of each stakeholder at each design life-cycle phase; how deep analysis of design will take place, ranging from physical ergonomics to cognitive levels in the context of use directed to Industry 4.0. Future research needs to express these success factors that deserve attention and emphasis in a comprehensive way to avoid research limitations and market failures in industry.

Another enabling success factor is the knowledge management of design data. The digital technologies—IoT, artificial intelligent, virtual and mixed reality—facilitate the design knowledge to be collected, organized, retrieved, and reused in an effective manner. This advantage in Industry 4.0 enables designers to facilitate the multidimensional approach in the design knowledge that ranges from physical stress, to physiological data, to social data: culture, human behaviour, emotion, and background. In addition to the technology, a well-established method to construct and manage design knowledge is worth considering in future research. The useful method in this case is to establish a knowledge platform that defines a collection of semantic commonalities derived from historical design ontology-based databases. This platform design enables a new stream of products and/or services to be developed in an efficient manner towards economics and manufacturability: design for variety and customization, the use of the existing design experience, and expertise that reduces design efforts and enhances collaborative working.

In addition to the success factors, 10 out of 43 case studies provide quantifiable outcomes. These results prove that the robustness and performance of the systems can be achieved with the applications of HCD in different aspects: human diversity, ergonomics, economics, manufacturability, and sustainability. A limited array of studies incorporates human diversity—human culture, society, background—to improve robustness and sustainability—which combine the human difference with the extended service life—of design solutions. In contrast, numerous studies enhance the robustness in human performance by ergonomics: avoidance of workplace risks and reduction in biomechanical workloads. This outcome also improves economics and manufacturability in terms of production performance: productivity, engineering time, and quality. Moreover, the engineering methods—design for product platform and family, design for multi-functionality and modularity alike—seek a common design platform that paves the way for manufacturability and economics: reduction in design effort, time-to-market for future generations of products and/or services. Beyond the engineering methods, future research needs to embrace the financial perspective to quantify and evaluate the economics of HCD, such as the cost–benefit analysis that can also be extended to the life-cycle cost analysis. However, each study limits its research scope in one of these aspects, which provides a pivotal research space for subsequent researchers, who should grasp these aspects in their research of HCD within a comprehensive approach. Besides, the rest of the case studies provide limited information about how their design proposals are effective in quantifiable ways, creating a need for future quantitative research rather than the qualitative approach. Regarding this research opportunity, it is also useful to make contributions to the creation of a design evaluation system oriented to the process of HCD. This design evaluation system has the following ultimate objectives: to evaluate how well the decisions and activities that are made during the design phases actually turn out, to monitor the design process, and to facilitate decision making on any potential breakdowns and pitfalls.

Other research efforts provide the design frameworks in different contexts of use: the supporting design frameworks that facilitate the design process in an effective manner and the collaborative design frameworks that promote the transdisciplinary and multidimensional approach. The former provides systematic design thinking—integrated design methods to avoid valueless reworks and activities towards economic sustainability—and possible ways to tackle different challenges—the complexity and uncertainty in the relationship between human and non-human actors—scattered across life-cycle design phases. The latter unfolds the common expertise of transdisciplinary teams to co-create value-in-use for customers and also generate benefits—financial and non-financial measures—for all network partners towards sustainability. These frameworks reflect perspectives of the common platform design and life-cycle cost analysis, which are useful considerations for future research to contribute to multi-objective HCD in a comprehensive way.

The minority of case studies have paid attention to experience-driven design with visualization techniques: design fiction with the video-illustrated prototype, and virtual and augmented reality. These case studies give inspirational examples of how digital technologies enrich the human experience, rather than physical real prototypes that are difficult to produce or interpret in abstract verbal descriptions. This approach examines future possibilities of new design that allow designers to comprehend the human experience and go beyond technical design towards the multidimensional approach, from technical to social aspects. In this respect, another interesting research domain would be exploring the possibility of making the best of the technologies in the age of Industry 4.0 to support the process of HCD. This direction of future research would be beneficial to fulfilling the limitations—namely RL4 in Table 8—that express different concerns: computational capability (Ceccacci et al., 2019; Chen et al., 2016; Leng & Jiang, 2017), data synchronisation (Lin, 2018; Peruzzini et al., 2019), and knowledge management (Fu et al., 2019; Grandi et al., 2020; Vanderhaegen, 2019; Zhu et al., 2015).

A limited range of studies put the perspective of human diversity towards the multidimensional approach that considers not only design artefacts but also the social aspects—background, age, gender, education, cultural influences, privacy management—in design. Lack of consideration of the difference between humans could thwart all research efforts and lessen potential benefits. This is particularly true in the context of population aging, which makes human diversity an essential consideration across diverse fields (Ahmadpour et al., 2019; Dankl, 2017; Lee & Coughlin, 2015). This phenomenon challenges manufacturing design in Industry 4.0, requiring a multi-objective methodology to capture diverse human factors. For example, Dalle Mura and Dini (2019) optimized ergonomics in assembly lines by proposing a multi-objective genetic algorithm capturing human factors: age, gender, weight, height, and skill. However, Katiraee et al. (2019) indicated that human differences regarding age and skill have been well studied in the literature, while few studies investigate other human aspects, including cognitive abilities. Therefore, future research on the topic should be ready to accommodate individualization in accordance with human diversity to encapsulate a new relationship between society and technology in the context of Industry 4.0.

Last but not least, the robustness of the research findings could be jeopardized if the identified limitations could not be alleviated. The majority of identified limitations are assigned to the experimental set-up conditions: low sample sizes, lack of fitting in target participants, lack of sound statistical studies, and other biased experimental aspects. There is also insufficient evidence of the extent to which these findings in one context can be applied and reproduced in other contexts. Future research would be trying to establish and enhance the robustness of research results by satisfying certain criteria for validity, such as the use of multiple sources of evidence, replication logic in multiple-case studies, and the well-established protocol of design for case study (Isaksson et al., 2020; Voss et al., 2002).

Throughout the value chain, the impact and increasing challenges of the transition to Industry 4.0 mean that integrating the role of humans is a part of the transition. It is going to attract more and more research efforts for the next decade, at least in the following five years. This is an opportunity to look back in a systematic manner on what the literature has achieved and the lessons it’s learnt, as summarized in the following points for the considerations of future research:

- *Research approach*: The fulfilment of the transdisciplinary and multidimensional HCD needs to be achieved through a systematic identification of stakeholder networks, levels of their involvement in each life-cycle design process, and design practice.
- *Research scalability and robustness*: The proposals of a design methodology should provide well-proven empirical results in well-validated case studies in varied contexts in which the individualization towards human diversity is taken into account.
- *Research performance*: A holistic approach is needed to make the best of Industry 4.0 technologies, facilitating the process of HCD in which both human and non-human actors are integrated towards human diversity, ergonomics, economics, manufacturability, and sustainability.
- *Research framework*: A new validated framework of HCD should take the points above into account and incorporate a well-rounded evaluation methodology to quantify the outcome of design activities across the life-cycle design phases. Besides, an interesting consideration in future research is to unify the relationships among the variants of HCD in order to embed them into the complete infrastructure of Industry 4.0.

These research schemes are challenging in a way that requires the increasing involvement of transdisciplinary collaboration in which researchers and industrial experts are brought together. This collaborative research is especially called in the phenomenon in which a transdisciplinary and multidimensional approach is required for a specific scientific topic (Chen & Duh, 2019; Hammer et al., 2018). This is also an approach for our next contribution.

## Conclusion

Active work on developing methods, exploring influencing factors, and proving the effectiveness and efficiency regarding HCD show the increasing awareness of human roles in Industry 4.0. However, numerous studies have been brought into existence, but then subsequently disconnected from other studies. As a consequence, the application of these studies in industry and research alike is not regularly adopted, and the array of studies is broad and expands in different directions without forming a coherent structure. This study is one of the unique attempts to bridge the gap between the literature characteristics and the lessons learnt derived from an expository of case studies of HCD in the context of Industry 4.0. In order to sufficiently cover the research topic and provide evidence with a minimal amount of subjectivity and bias, this research performs SLR in which a special unit of analysis is given to the case studies, delivering the contributions in three ways. First, the approach to HCD claims to be transdisciplinary and multidimensional, which is evidenced by the overall literature characteristics: increasing research interest across disciplines and industries in different levels of analysis—product, workstation, company, and society.

Secondly, the transdisciplinary and multidimensional approach is also reflected by the in-depth review of case studies: the emerging trend, the design methods and lessons learnt. The review of the 43 case studies unfolds the emerging research themes—HCD, PSS, UCD—that deal with the challenges of personalization, servitization, and sustainability in the context of Industry 4.0. This phenomenon also leaves research space for the other concepts—HRC, HioTL, HMI—in smart manufacturing in the form of empirical research. Besides, the in-depth review also captures the wide range of design methods that are categorized in the four generic groups—discovery, clean-up, engineering, experiment—to tackle different problems scattered across different life-cycle design phases. Furthermore, the implementation of these design methods is also facilitated with the support of digital technologies: virtual and mixed reality, eye-tracking systems, digital modelling and simulation for virtual workplaces, IoT solutions, artificial intelligent. The variety in both quantitative and qualitative design methods associated with the supporting technologies expresses the necessity of the transdisciplinary and multidimensional approach for comprehending the methods in their proper context of use towards human diversity, ergonomics, economics, manufacturability, and sustainability. Therefore, for better adaption to the challenges, it is worth having cross-disciplinary collaborative research and/or improving the transdisciplinary skill sets of researchers and practitioners. This fact is further emphasized by the lessons learnt that dig into what the literature has achieved. The “Appendix” (Table 11)—which functions as a useful reference for the design of case studies—expresses the most important facts about the 43 case studies, resulting in the lessons learnt. These lessons learnt encapsulate various research results associated with limitations that are captured and harmonized in homogeneous groups: six groups of research results and four groups of research limitations. The research results are categorized into six groups: exploration of design success factors, achievement of engineering objectives, provision of supporting design frameworks, validation of the effect of human diversity on design, provision of transdisciplinary frameworks, and visualization of design scenarios. Different studies concentrate partially on their own expected results, which highlights a useful reference for future research that expresses both the transdisciplinary and multidimensional approach towards human diversity, ergonomics, economics, manufacturability, and sustainability in a comprehensive way. Besides, it is worth acknowledging the limitations—limited statistical power in result validation, lack of generalizability of research findings, further requirements of the supporting methods, lack of validation of the effectiveness—to enhance the robustness of the research findings. This will inspire research applications to both industry and research.

Third, the opportunities for future research regarding HCD in the context of Industry 4.0 are also provided to advance the research contributions in the coming years through the adoption of the lessons learnt from the previous works. Despite the rigor, relevance and expanse of this study, there are acknowledged limitations. Primarily, we applied the strict protocol of SLR with which some relevant papers might be overlooked. To minimize this, we searched eight databases to ensure a sufficient number of papers relevant to this topic to compensate for the missed papers—missed due to less relevance—by supplementing more relevant papers. Furthermore, we limited the papers to only peer-reviewed journal articles as a means to guarantee the quality of the publications. We also acknowledge that the selection of the topic, definition of search terms, and interpretation of the results are inseparable from our previous knowledge on the topic. Lastly, we assume that considerable knowledge resides among practitioners’ experience and the grey literature.

The particular interest in this topic is the question of how to take advantage of literature, overcome its own acknowledged limitations, and advance research contributions in the body of knowledge. The first two questions are provided in this study, and the last one can be achieved by collaborative research in which transdisciplinary and cross-sectorial research centres and industrial partners join forces to contribute to a comprehensive common understanding of HCD in the transdisciplinary and multidimensional approach towards human diversity, ergonomics, economics, manufacturability and sustainability. This is also the approach for our next contribution to the field of HCD.

## Data Availability

Not applicable.

## Appendix

See Tables 9, 10 and 11.

**Table 9:** Description of research databases

No	Database Name	Discipline	Description
1	Web of Science	Sciences, social sciences, arts, and humanities	Provides subscription-based access to multiple databases that provide comprehensive citation data for many different academic disciplines. It is currently maintained by Clarivate Analytics
2	Scopus	Life sciences; social sciences; physical sciences; health sciences	Provides the citation database of peer-reviewed literature: scientific journals, books and conference proceedings. From Elsevier
3	ScienceDirect	Physical sciences and engineering, life sciences, health sciences, social sciences and humanities	Provides subscription-based access to a large database of scientific and medical research. From Elsevier and related publishers
4	Emerald Publishing Limited	Management, business, education, library studies, health care, and engineering	Provides academic journals, books, and book series. Formerly MCB UP Ltd, the publisher changed its name to Emerald in 2002
5	SpringerLink	Science, technology, medicine, business, transport and architecture	Offers electronic and printed literature in different products: journals, books, series, protocols, reference works, and proceedings. From Springer plus journals from the publisher Wolters Kluwer
6	Engineering Village	Engineering	Provides integrated access to specialist databases as an information portal for engineering, applied science and technology. It includes Compendex and the Patents Office of the United States database
7	SAGE	Social sciences & humanities; health, life & biomedical sciences; engineering and physical sciences	Provides an independent, academic and professional publisher of different products, ranging from books, journals, online courses, etc.… SAGE has been part of the global academic community since 1965
8	EBSCO	Physics, electrical and electronics engineering, computers and control, information technology, manufacturing and production engineering	Provides comprehensive databases, ranging from general reference collections to specially designed and subject-specific databases for public, academic, medical, corporate and school libraries

**Table 10:** Adopted search syntax for each database

No.	Database name	Date	Search syntax *[**Search: by title, abstract, and keywords]*
1	Web of Science	21, June 2020	(TS = (("human centered design" OR "human centred design" OR "user centered design" OR "user centred design" OR "user experience design" OR "user oriented design" OR "human oriented design" OR "experience design" OR "service design" OR "interaction design") AND ("Industry 4.0" OR "industrie 4.0" OR "Cyber physical system*" OR "Cyber physical production system*" OR "smart manufacturing" OR "future manufacturing" OR "digital manufacturing" OR "smart factory" OR "future factory" OR "digital factory"))) AND LANGUAGE: (English) AND DOCUMENT TYPES: (Article)
2	Scopus	21, June 2020	TITLE-ABS-KEY (("human centered design" OR "human centred design" OR "user centered design" OR "user centred design" OR "user experience design" OR "user oriented design" OR "human oriented design" OR "experience design" OR "service design" OR "interaction design") AND ("Industry 4.0" OR "industrie 4.0" OR "Cyber physical system*" OR "Cyber physical production system*" OR "smart manufacturing" OR "future manufacturing" OR "digital manufacturing" OR "smart factory" OR "future factory" OR "digital factory")) AND (LIMIT-TO (DOCTYPE, "ar")) AND (LIMIT-TO (LANGUAGE, "English"))
3	Science Direct	06, June 2020	3.1. ("human centered design" OR "human centred design" OR "user centered design" OR "user centred design") AND ("Industry 4.0" OR "industrie 4.0" OR "Cyber physical system" OR "Cyber physical production system") 3.2. ("human centered design" OR "human centred design" OR "user centered design" OR "user centred design") AND ("smart manufacturing" OR "future manufacturing" OR "digital manufacturing") 3.3. ("human centered design" OR "human centred design" OR "user centered design" OR "user centred design") AND ("smart factory" OR "future factory" OR "digital factory") 3.4. ("user experience design" OR "user oriented design" OR "human oriented design" OR "experience design" OR "service design" OR "interaction design") AND ("Industry 4.0" OR "industrie 4.0" OR "Cyber physical system") 3.5. ("user experience design" OR "user oriented design" OR "human oriented design" OR "experience design" OR "service design" OR "interaction design") AND ("Cyber physical production system") 3.6. ("user experience design" OR "user oriented design" OR "human oriented design" OR "experience design" OR "service design" OR "interaction design") AND ("smart manufacturing" OR "future manufacturing" OR "digital manufacturing") 3.7. ("user experience design" OR "user oriented design" OR "human oriented design" OR "experience design" OR "service design" OR "interaction design") AND ("smart factory" OR "future factory" OR "digital factory")
4	Emerald	06, June 2020	(content-type:article) AND ("human centered design" OR "human centred design" OR "user centered design" OR "user centred design" OR "user experience design" OR "user oriented design" OR "human oriented design" OR "experience design" OR "service design" OR "interaction design") AND ("Industry 4.0" OR "industrie 4.0" OR "Cyber physical system*" OR "Cyber physical production system*" OR "smart manufacturing" OR "future manufacturing" OR "digital manufacturing" OR "smart factory" OR "future factory" OR "digital factory")
5	Springer Link	21, June 2020	("human centered design" OR "human centred design" OR "user centered design" OR "user centred design" OR "user experience design" OR "user oriented design" OR "human oriented design" OR "experience design" OR "service design" OR "interaction design") AND ("Industry 4.0" OR "industrie 4.0" OR "Cyber physical system*" OR "Cyber physical production system*" OR "smart manufacturing" OR "future manufacturing" OR "digital manufacturing" OR "smart factory" OR "future factory" OR "digital factory")
6	Engineering Village	21, June 2020	((((("human centered design" OR "human centred design" OR "user centered design" OR "user centred design" OR "user experience design" OR "user oriented design" OR "human oriented design" OR "experience design" OR "service design" OR "interaction design") AND ("Industry 4.0" OR "industrie 4.0" OR "Cyber physical system*" OR "Cyber physical production system*" OR "smart manufacturing" OR "future manufacturing" OR "digital manufacturing" OR "smart factory" OR "future factory" OR "digital factory")) WN KY)) AND (({ja} WN DT) AND ({english} WN LA)))
7	SEGA Journals	10, June 2020	for [[All "human centered design"] OR [All "human centred design"] OR [All "user centered design"] OR [All "user centred design"] OR [All "user experience design"] OR [All "user oriented design"] OR [All "human oriented design"] OR [All "experience design"] OR [All "service design"] OR [All "interaction design"]] AND [[All "industry 4.0"] OR [All "industrie 4.0"] OR [All "cyber physical system"] OR [All "cyber physical production system"] OR [All "smart manufacturing"] OR [All "future manufacturing"] OR [All "digital manufacturing"] OR [All "smart factory"] OR [All "future factory"] OR [All "digital factory"]] Within Research Article
8	EBSCO	13, June 2020	("human centered design" OR "human centred design" OR "user centered design" OR "user centred design" OR "user experience design" OR "user oriented design" OR "human oriented design" OR "experience design" OR "service design" OR "interaction design") AND ("Industry 4.0" OR "industrie 4.0" OR "Cyber physical system*" OR "Cyber physical production system*" OR "smart manufacturing" OR "future manufacturing" OR "digital manufacturing" OR "smart factory" OR "future factory" OR "digital factory") Limited to: English, Peer-Reviewed, Academic Journals

**Table 11:** Summary of case study results according to literature

References	Design concepts	Context of case study	Objective	Design method	Evaluation methods	Research results^a^ (RR)	Result limitations^a^ (RL)
Schulze et al. (2005)	Human-centred design	Design for the computer-aided technology systems with respect to the production planning process at Daimler Chrysler AG	Identify requirements and success factors of a human-centred and computer-aided planning tool application	Iterative design *(participatory design, prototyping)*	Questionnaires *(8 planers for usability)* Interviews Scenario observation *(3 participants in a click-test scenario)*	(1) Large and time-consuming software projects for complex domains can be successful in applying human factors (2) Interdisciplinary and participative development of complex engineering applications are vital	(1) Limited information on determination of the required sample size (power) (2) Lack of generalizability
Lee and Abuali (2011)	Product-service systems	Design for value propositions at a company that specializes in the pressure-sensitive technology and self-adhesive solutions for consumer products	Demonstrate the pathway to the development of dominant design through effectively utilizing the proposed key tools	Quality function deployment *(combined with application mapping, novel innovation matrix)*	Undefined	(6) Provide the systematic thinking and dominant design of product–service systems that reply on the integration of novel tools	(2) It is unclear the findings are transferable to real environments (3) Lack of validation on the model´s effectiveness (4) In need of automation of the proposed tools
X. Wu et al. (2013)	Human-centred design	Design for a multi-function bike—a scooter and a tricycle—that can be used for both gliding and riding that fit the body size of children (aged 5–12)	Validate innovative product design using a rational multi-function method through functional superposition, transformation, and technical implementation	Human factors and ergonomics	Undefined	(1) The design innovates the design of children’s bikes and extends the service life, which introduces energy saving features and develops intensive concepts	(2) Unreal industrial implementation (3) Lack of validation on the model’s effectiveness
Zhu et al. (2015)	Product-service systems	Design for the knowledge-based support system in service operations of an aircraft engine	Focus on developing a knowledge-based support system for PSS design, and generating the PSS solutions to customer requirements	Quality function deployment Mathematical models *(analytical network process)* Iterative design *(prototyping)*	Undefined	(2) Customer requirements are considered during the design phase Knowledge could be identified and reused easily during PSS activities	(3) Lack of validation on the model´s effectiveness (4) In need of software development of the proposed tools
D. Chen et al. (2016)	Human-centred design	Design for pan-ethnic-group products with collection of various types of pictures that are assessed by emotional semantic feedbacks from user perspectives	Validate the proposed method of pan-ethnic-group products based on the gene clustering method and emotional semantic analysis	Mathematical models *(fuzzy set theory, principal component analysis)*	Undefined	(1) Improve the agility of the product design process in Industry 4.0 (2) User percepts are considered at an early stage of product design	(2) Unreal industrial implementation (3) Lack of validation on the model´s effectiveness
L. Wu et al. (2016)	Human–machine interface	An experimental study on an eye tracking in LED manufacturing systems to measure three levels of interface complexity on user experience	Investigate the relationship between user experience and interface design with respects to the complexity levels and user background	Human factors and ergonomics	Questionnaires *(subjective participants’ responses with 19 experts and 19 novices)* Statistics *(hypothesis testing)*	(3) User experience is significantly affected by the factors, including the interface complexity and user background	(1) There is limited information on the determination of required sample size and the participants are all from the same company. Therefore, this study has a potential bias
R. Y. Chen (2016)	User-centred design	Design for complex interactions and experiential design system with fuzzy dual experience-based design on consumer electronic products	Illustrate the usability of the proposed model and its sensitivity analysis	Mathematical models *(fuzzy decision tree, fuzzy cognitive map, analytic hierarchy process)*	Mathematical models *(linear programming)*	(1) Find the optimal profit of the design quality in relationship between market price and experience’s product value (2) Both the engineer’s experience and customer’s experience are important	(2) It is unclear the findings are transferable to real environments (4) The quality of the model will depend on the input case data as well as computational capability
Song et al. (2016)	Human–machine interface	A user study using a paper prototype (wireless connections) to investigate how information of interface design are interpreted and evaluated by users	Assess whether the revised interface, with the proposed method, helps users accomplish their tasks	Iterative design *(research through design)*	Scenario observation *(10 non-expert users, and 5 participants for the printer-iPad connection)*	(6) Provide a design framework for interpreting and resolving complex user interactions	(1) Limited statistical power (2) Lack of generalizability of results to all applications
Jung et al. (2017)	User-centred design	Design for mitigation of the elderly’s growing fatal accidents by considering concept usability and body conditions in the different countries: South Korea, United States of America, and the United Kingdom	Understand what kinds of driving problems elderly drivers have and demonstrate how new system concepts could be developed	Iterative design *(interviews, questions, focus groups, observation)*	Questionnaires Interviews *(60 elderly people)*	(3) Provide different perspectives to anticipate safe and usable solutions of elderly drivers in hopes of mitigating accidents	(1) Limited statistical power (2) Lack of generalizability of results to all other regions
Kymäläinen et al. (2017)	User-centred design	Design for new interaction methods and ambient intelligence of an oil refinery factory focusing on the production of advanced and low-emission traffic fuels in Finland	Demonstrate the future-oriented user experience research through the method of video-illustration	Iterative design *(science fiction prototyping)*	Questionnaires Interviews *(23 operators)*	(4) Show the reflection of creation process and how the experience-driven with video-illustrated prototype is evaluated	(1) Encompass degrees of subjectivity and rely on knowledge, judgment and projection (2) Lack of generalizability of results to all applications
Leng and Jiang (2017)	Product-service systems	Design for a reference model of the historical service cases from a manufacturing context of a special printing machinery enterprise group in a China Technology Zone	Aim at illustrating the practicality of the proposed approach for new product and service development	Mathematical models *(fuzzy set theory and quotient space)*	Mathematical models *(Fuzzy tolerance quotient spaces)*	(2) The proposed approach is suited to be applied on real-world data to extract and reuse the “best practice” knowledge from historical cases	(4) The quality of the model will depend on the input case data as well as computational capability
Ma et al. (2017)	User- centred design	Design for customer requirements translated into product specifications in the context of high-speed train	Verify the rationality and feasibility of the multidisciplinary requirement modelling	Iterative design *(interviews)* Quality function deployment	Undefined	(5) Guide the multidisciplinary collaborative design that can be inversely mapped into each disciplinary	(3) Lack of validation on the model´s effectiveness (4) ‘Manual’ application of the proposed methodology
Pacaux-Lemoine et al. (2017)	Human- centred design	Design for human integration in intelligent manufacturing systems in which two technonogy-centred approaches—without and partial human involvement—are compared with HCD	Validate the proposed framework of HCD on production demonstrator Focus on the issue of human-system cooperation	Human factors and ergonomics	Questionnaires *(1 survey for the workload and acceptability evaluation)* Performance comparison	(1) Improve the global production objectives Facilitate human operator workload	(1) Limited statistical power (2) Unreal industrial implementation
Boy (2018)	Human- centred design	Design for human-systems integration in which the Mars 500 experiment was carried out	Understand the long-term isolation in a limited room space with different cultural crews of volunteers	Iterative design *(participatory design)*	Undefined	(3) Three major issues are elicited: time effects; cultural influences; and individual differences	(3) Lack of validation on the model´s effectiveness
Kaasinen et al. (2018)	Human- centred design	Design for a comprehensive coverage of maintenance work in the context of Industry 4.0 with four industrial demonstrators	Illustrate how industrial maintenance work could benefit from knowledge-sharing solutions based on Industry 4.0	Iterative design *(field studies, interviews, observation)*	Questionnaires *(2 maintenance technicians)* Scenario observation Interview	(4) Illustrate the user experience of future maintenance work that shares knowledge with peers and make reports effortless	(1) Limited statistical power (2) Problematic design and technology applications related to wearable devices
Lin (2018)	User- centred design	Design for a user experience-based design approach with a glass recycling company in Taiwan to enhance recycling incentives and empower Industry 4.0	Verify the proposed approach’s practical feasibility	Iterative design (*UNISON framework of data-driven innovation, questionnaires*)	Undefined	(2) Understanding users’ preferences and actual needs is critical (6) Enable follow-up experiments for reference and modification to facilitate understanding of changing user preferences	(3) Lack of validation on the model´s effectiveness (4) Focus only short-term user data that are required to be updated every season for new insights
Mazali (2018)	User-centred design	A study on the links among digital society, digital culture and Industry 4.0 in the context of an Italian plant where high-speed trains are produced	Examine the change that workers are subject to and along with the work organization in smart digital factories	Iterative design *(interviews)*	In-depth interviews *(40 interviews were conducted with managers and middle managers)*	(2) User-mental models and sensemaking need to be updated with social and organizational contexts that involve stakeholders and new roles of intelligent systems in workflows	(1) Limited statistical power (2) The extension to additional companies and sectors is required to achieve a generalization
Mostafazadeh Davani et al. (2018)	Human–machine interface	Design for a 3D based meta user interface that is specifically developed to support interaction with ambient intelligence systems	Illustrate the designed meta-UI that can increase usability of the human-meta system interaction	Iterative design *(focus groups, GUI prototyping, cognitive walkthrough)*	Questionnaires *(6 participants with the System Usability Scale questionnaires)*	(6) The proposed meta-UI can support the usability of human-meta system interaction	(1) Limited statistical power (2) Lack of generalizability of results to all applications
Mourtzis et al. (2018)	Product-service systems	Design for the quantification of PSS customization complexity in the context of machining industry that is exemplified with 30 product-service alternatives	Aim at evaluating the different alternatives of PSS in terms of complexity	Mathematical models *(geometric vectors, linear algebra)*	Mathematical models *(Vector Analysis in the Euclidian space*)	(2) The competitiveness and sustainability of enterprises can be maintained by monitoring their PSS throughout their life cycle (6) Estimate the complexity of different design alternatives towards the selection of PSS	(2) The model does not address other factors, such as need/nature of different industrial companies (4) Require development of a software application for computation
Pezzotta et al. (2018)	Product-service systems	Design for PSS along their entire life cycle in the industrial context of a mould-making B2B Greek small-medium size enterprise	Develop and validate the proposed methodology of Product Service System Lean Design Methodology	Iterative design *(traditional brainstorming, focus groups, cognitive walkthrough and Wizard of Oz, prototyping)*	Face-to-face workshop	(6) Create PSS that are customer driven, economically sustainable in the long term and avoid valueless reworks and activities	(2) It is unclear the findings are transferable to real environments
Richert et al. (2018)	Human–robot collaboration	Design for the hybrid human–machine team (socializing with robots) by using Cinema 4D and Unreal Engine in which participants are randomly assigned to four different set-up conditions	Analyse hybrid cooperation and team building in a controlled setting in which a production hall and a robotic teammate are built	Human factors and ergonomics	Questionnaires *(112 out of 153 student responses)* Statistics *(hypothesis testing)*	(2) The hybrid team humanoid appearance might be a more stable condition for different personality types and vice versa for the case of machine-like robots	(1) Limited statistical power and the participants are not intended end-users (2) It is unclear the findings are transferable to real environments
Wang et al. (2018)	Human-centred design	Design for smart clothing prototypes embedded with micro-sensors and light-emitting diodes (LEDs) functions to enhance human emotional expression	Validate a methodology bridging the gap between human emotions and wearable technologies for interactive fashion innovation	Iterative design *(prototyping)* Kansei engineering	Questionnaire *(emotional survey)* Scenario observation *(34 participants with Kansei method)*	(2) The functionality should synchronize with the requirements of human emotional expression to stimulate the emotional response	(1) The participants’ wearing experience time is limited, and the evaluation is mainly subjective
Anke (2019)	Product-service systems	Design for a meta-model and web-based tool to combine the financial assessment with service design that embraces collaborative teamwork	Evaluate the tool in which multiple teams carry out the design and evaluation of smart services	Iterative design *(a web-based tool prototype)*	Questionnaires *(30 participants)*	(5) Provide interdisciplinary teams a tool-based structuring support for the design and evaluation of smart services	(1) Limited statistical power (4) Further clarification of conditions under which the usage of the tool becomes more effective is required
Caputo et al. (2019)	Human- centred design	Design for ergonomics to preventively solve ergonomic risks by simulation with a ‘Fiat Panda’ assembly line	Validate an appraisal of workplace design towards preventive ergonomics by virtual workplace simulation	Human factors and ergonomics	Mathematical models (Simulation *with data from assembly tasks simulation of Digital Human Models)*	(1) It is possible to preventively solve ergonomic risks during the design phase	(2) Unreal industrial implementation
Ceccacci et al. (2019)	Human- centred design	Design for ergonomics to understand the relationship among ergonomics, safety and risk classification in the context of manual assembly of the Cooker Hood Line	Demonstrate the proposed multi-path methodology to support the application of ergonomic risk management in practice	Human factors and ergonomics	Ergonomic analysis Physical ergonomic assessment (RULA) *(2 participants)* Interviews *(8 participants)*	(1) Provide the definition of crucial risk factors and selection of proper ergonomics assessment associated with measurement tools	(1) Limited statistical power (2) Without taking into account psychosocial stressors (4) Manual application of the proposed methodology
Fu et al. (2019)	Human- centred design	Design for a model of cyber-physical public design to explore the three dimensions: residents’ information exchange, friends’ interaction and personal emotion	Illustrate the model of data collection on residents’ lives, improve and rebuild the urban communities to meet the needs of the public	Iterative design *(participatory design)*	Questionnaire *(8 participants *via* living lab experiment)* Scenario observation	(2) User data can improve the interaction experience sustainably Integrating virtual and real world plays an important role in the construction of smart cities	(1) Limited statistical power (2) Unreal industrial implementation
Grieger and Ludwig (2019)	Product-service systems	Design for digital service in providing parking spots of a major Swedish spare-part supplier	Evaluate the proposed conceptual reference framework of digital service conceptualization in the early design phase	Iterative design *(five guideline-supported interviews)*	Interviews *(3 OEM employees and 1 IT external industry expert)* A case study workshop	(2) Support the early development stage by giving a structure and a customer-centric direction	(1) Limited statistical power (4) A set of supplementary tools for service development is required
Haber and Fargnoli (2019)	Product-service systems	Design for the product and service integration to achieve functional results with offering’s value at a manufacturer that produces hemodialysis devices associated with services	Validate the proposed model’ application	Iterative design *(questionnaires, interviews)* Quality function deployment Mathematical models *(Thurstone’s Law of Comparative Judgments)* Kano model	Undefined	(6) Facilitate the company to collect information in the case of incomplete answers to surveys and questionnaires, which provides a practical method to handle the uncertainty	(2) The “end” users of the equipment (i.e. the patients) is not considered (3) Lack of information in effectiveness validation on the proposed solutions
Harwood et al. (2019)	Product-service systems	Design for a diegetic prototyping methodology to investigate service innovations that reflect future uses of new and emerging technologies	Provide an example of IoT applications, illustrate the central proposed tenets, and identify key issues	Iterative design *(science fiction prototyping)*	Questionnaires *(1,200 respondents)*	(4) Facilitate visualization that examines future possibilities of service innovations-in-use by overcoming abstract verbal descriptions of new technologies	(1) Limited statistical power with certain degrees of subjectivity, judgment and projection
Hoe (2019)	Human- centred design	A study on the digitalization journey in the context of community hospital and social service agency that deliver holistic care for seniors	Illustrate the relevant five disciplines in the digitalization journey: personal mastery, mental models, shared vision, team learning and system thinking	Empirical experiments	Undefined	(2) The patient centric model is a key of success (3) Alleviate the issue of overcrowding in hospitals, medication non-compliance and social isolation of seniors	(3) Lack of information in effectiveness validation on the proposed solutions
Kong et al. (2019)	Human-in/on-the-loop	A study on design consideration for industrial wearable systems addressed by three world-leading development groups	Review and analyse academic progresses to provide insights into the past, present and future of industrial wearable systems	Iterative design *(questionnaires)*	Questionnaires Interviews Workshops *(25 company representatives)*	(5) Structure a new framework with three aspects: design scalability, reconfigurability and robust architecture	(1) The propositions require testing by further quantitative methods
Peruzzini et al. (2019)	Human- centred design	Design for assembly stations in collaboration with CNH Industrial to validate the use of the multimodal approach in workplace and product design (tractors’ cabin)	Validate the proposal with the application of VR technologies that allow for interactive design of products towards HCD	Iterative design *(Norman’s interaction design model)* Human factors and ergonomics	Questionnaires *(4 novices and 4 experts)* Scenario observation	(2) Have a precise and objectified feedback about interaction design before the product/process realization to improve the system design or re-design	(1) Limited statistical power (4) Costs and Complexity of the set-up, and the need of multiple data collection and synchronization
Prinz et al. (2019)	Human-in/on-the-loop	Design for a graphical I4.0-enabled engineering method that is prototyped and implemented in three manufacturing scenarios: automated station, manual workplace, transport system	Evaluate the proposed method with case studies in which participants are asked to solve multiple engineering tasks	Iterative design *(prototyping)* Business Process Modelling and Notation	Questionnaires *(10 participants)* Performance comparison Statistics *(hypothesis testing)*	(1) Significantly outperform the conventional method in terms of required engineering times and subjective ratings	(1) Limited information on determination of the required sample size (power)
Turetken et al. (2019)	Product-service systems	Design for an integral component of a business engineering framework that introduces the service-dominant business model radar in the mobility domain	Evaluate the proposed model for its validity and utility	Iterative design *(focus groups)*	Workshops (15 *workshops with 161 practitioners designing blueprints for 21 new business models)* Questionnaires *(36% response rate)*	(2) Take advantage of various capabilities of multiple parties to offer value to customers (5) Develop a business modelling that motivates a collaborative approach	(1) Limited statistical power (2) It is unclear the findings are transferable to real environments
Vanderhaegen (2019)	Human-in/on-the-loop	Design for human–machine systems on the rail flow control with the experiments: automation‑supported human and human‑supported automation	Test the proposed concept with two case studies on rail flow control in relation to pedagogical learning supports	Empirical experiments	Questionnaires *(56 students)* Scenario observation	(2) Adapt the online learning process to individual requirements and limits	(1) Limited statistical power
Witschel et al. (2019)	Product-service systems	A case study on the mechanism behind digital capabilities toward digitization in large German companies	Explore causal mechanisms and the derivation, test and develop theoretical constructs	Iterative design *(interviews, theory-guided approach for text analysis)*	Interviews *(15 semi-structured interviews with executive and senior managers from 8 larger companies)*	(2) Digital capabilities are only effective, if there is an alignment between strategy, organizational and leadership mindset	(2) Lack of generalizability of results to all types of industries and firms
Wojtynek et al. (2019)	Human–robot collaboration	Design for a collaborative robot combined with Plug-and-Produce system where participants perform a flexible work-cell setup	Evaluate both the technical aspects: the usability of the smart system, and human factors	Human factors and ergonomics Empirical experiments	Questionnaires *(17 participants university spectrum and laboratory co-workers)*	(1) Participants do not excessively extend physically and mentally during the experimental scenarios	(1) Limited statistical power
Adrodegari and Saccani (2020)	Product-service systems	A study on a servitization maturity model that aims at assessing and positioning companies in the context of a medium machine-tool builder and a forklift truck firm	Exemplify how the proposed model supports companies in identifying and bridging the gaps in order to deploy a servitized model	Iterative design *(a full-day meeting with the top managers)*	Interviews	(6) Support SMEs in the servitization journey, and help them bridge the distance with large companies	(2) The extension to additional companies and sectors is required to achieve a generalization
Grandi et al. (2020)	Human- centred design	Design for serviceability with mixed reality to evaluate two different use cases that support human-centred system development in tractor design	Demonstrate a way in which the proposed approach can support human-centred system development	Human factors and ergonomics	Questionnaires *(1 user)* Performance comparison *(on ergonomic aspects with the support of MR tools)*	(2) Digital technologies can provide both the physical and digital worlds that allow to foresee interactions to predict and fix design criticalities	(1) Limited statistical power
Gualtieri et al. (2020)	Human- centred design	Design for a robotic collaborative workstation of a wire assembly line that interacts with an operator in an ergonomic and efficient way	Demonstrate improvements in the ergonomics in terms of productivity and reduction of biomechanical overload	Human factors and ergonomics	Ergonomic analysis Physical ergonomic assessment (RULA) *(2 participants)*	(1) Perform an accurate assessment of ergonomics in the early design phase Improve productivity and reduce biomechanical overload	(1) Limited statistical power (2) Further improvement is required to replace the RULA method that is more suitable for preliminary postural assessment
Van Acker et al. (2020)	User-centred design	A study on the understanding of employee objections to wearing gauges of wearable mental workload as leveraged by Industry 4.0	Test a hypothesis of how user acceptability of technology depends on the technology’s goals and implementation context of the wearing gauges	Iterative design *(questionnaires)*	Questionnaires *(150 participants for pilot study and 350 responses for pre-registered study with gender analysis)* Interviews Statistics	(3) Technology readiness, gender, education, and possibly proper privacy management are found to play a crucial role	(1) Limited information on determination of the required sample size (2) The replication of research result was not successful
van Lopik et al. (2020)	User-centred design	Design for knowledge sharing between expert and novice workers during the phone repair task with an augmented repair training application	Implement and evaluate the proposed application	Iterative design *(iterative refinement through usability testing and feedback)* Human factors and ergonomics Empirical experiments	Questionnaires *(5 participants)*	(2) Engage management is highly recommended (6) Help small enterprises explore the value of AR whilst causing minimal disruption and limited financial investment	(1) Limited statistical power (4) The need for data protection, maintenance and storage were not directly addressed
Zhang et al. (2020)	Product-service systems	Design for an environmental model-driven interaction of smart products through-life design of service and maintenance, which is virtual and supported by integrated software solutions	Evaluate the model´s implementation feasibility in an industrial case study of smart high-speed train	Iterative design *(Norman’s interaction design model)*	Undefined	(2) Address intelligent interactions with external environments and relevant stakeholders (6) Map out the interaction requirements between the smart product and other interaction elements	(3) Lack of information in effectiveness validation on the proposed solutions (4) Required ability to achieve the computerization of the design framework

^a^The extracted items are numbered and categorised in the same corresponding numbered groups (RR1, RR2, RR3, RR4, RR5, RR6, RL1, RL2,RL3, RL4) in accordance with their affinity
